# Progranulin-driven lysosomal acidification facilitates exocytosis of PHEV-hijacked lysosomes for viral release

**DOI:** 10.1128/mbio.02903-25

**Published:** 2025-11-25

**Authors:** Zhenzhen Wang, Yuzhu Chen, Wenqi He, Yuzhu Liu, Yubo Jiao, Gaili Wang, Jiyu Guan, Kui Zhao, Qiaoling Zhang, Feng Gao, Zi Li, Yungang Lan

**Affiliations:** 1 State Key Laboratory for Diagnosis and Treatment of Severe Zoonotic Infectious Diseases, Key Laboratory for Zoonosis Research of the Ministry of Education, Institute of Zoonosis, and College of Veterinary Medicine, Jilin University 623814, Changchun, China; 2 Jilin Academy of Animal Husbandry and Veterinary Medicine, Changchun, Jilin, China; Dartmouth College, Hanover, New Hampshire, USA

**Keywords:** progranulin, lysosomal acidification, lysosomal exocytosis, porcine hemagglutinating encephalomyelitis virus, betacoronaviruses, central nervous system

## Abstract

Porcine hemagglutinating encephalomyelitis virus (PHEV) hijacks lysosomes, repurposing them as vehicles for viral release by disrupting their function. Progranulin (PGRN), a lysosomal glycoprotein essential for lysosomal regulation, plays a pivotal role in this process. This study demonstrates that PHEV infection reduces full-length PGRN levels while enhancing its lysosomal targeting. Notably, PGRN knockout mice exhibit resistance to PHEV infection, implicating PGRN in PHEV-induced lysosomal dysfunction and viral release. Mechanistically, PHEV infection-induced enhancement of PGRN lysosomal trafficking depends on the concurrent harnessing of two distinct pathways for lysosomal delivery of PGRN. This enhanced targeting involves increased vacuolar-type ATPase recruitment, intensifying lysosomal acidification and triggering Arl8b-dependent lysosomal exocytosis, facilitating viral release. Furthermore, *in vivo* knockdown of neuronal PGRN expression suppresses PHEV transmission within the central nervous system. These findings underscore the essential role of PGRN-mediated lysosomal acidification in driving lysosomal exocytosis, thereby contributing to PHEV release and neural dissemination during infection. These insights provide a foundation for targeting PGRN expression and lysosomal trafficking as a potential therapeutic strategy to mitigate PHEV infection and its neural dissemination.

**IMPORTANCE:**

Betacoronaviruses exploit the lysosomal exocytic pathway for cellular egress through diverse mechanisms, often leading to lysosomal dysfunction. Porcine hemagglutinating encephalomyelitis virus (PHEV), a neurotropic porcine betacoronavirus, relies on lysosomal acidification for cell egress, in contrast to other betacoronaviruses, such as severe acute respiratory syndrome coronavirus 2 and mouse hepatitis virus, which utilize deacidified lysosomes for release. Progranulin (PGRN), a lysosomal glycoprotein essential for regulating lysosomal function, has emerged as a critical player in this process. Here, we demonstrate that PHEV infection enhances PGRN lysosomal trafficking, with PGRN knockout mice exhibiting resistance to PHEV infection. Our findings reveal that PGRN expression and lysosomal targeting drive PHEV-induced lysosomal acidification, facilitating Arl8b-dependent lysosomal exocytosis and promoting viral egress. These results underscore the pivotal role of PGRN in lysosomal dysfunction and viral egress, warranting further investigation into its regulatory function during cellular egress of other betacoronaviruses.

## INTRODUCTION

Betacoronaviruses are positive-stranded, enveloped RNA viruses that cause a wide range of illnesses in humans and animals, including domestic and wild species (1). Among them, porcine hemagglutinating encephalomyelitis virus (PHEV) poses a significant threat to the pork industry. First isolated in 1962 from suckling piglets in Canada with neurological disorders, PHEV belongs to the *Betacoronavirus* genus, Coronaviridae family, Coronavirinae subfamily, and Nidovirales order (2, 3). In pigs, PHEV infection typically manifests as encephalomyelitis, vomiting, wasting disease, and influenza-like symptoms (4, 5). With no commercial PHEV vaccines available, neonatal protection against PHEV-associated diseases depends on lactogenic immunity (6). Notably, studies using mice or neuro-2a (N2a) cells suggest that PHEV may serve as a model for investigating betacoronavirus-associated olfactory and taste disorders and neurodegenerative disease mechanisms (7, 8). Despite these advances, a deeper understanding of the PHEV life cycle, including mechanisms of virus entry and release, remains critical for vaccine development and for elucidating PHEV-associated neurological diseases.

Lysosomes, membrane-bound organelles, play central roles in cellular processes such as endocytosis, phagocytosis, and autophagy, maintaining a highly acidic environment (pH 4.5–5.5) through the activity of vacuolar-type ATPase (V-ATPase) (9). V-ATPase, a membrane transporter, drives proton transport into lysosomes via ATP hydrolysis, sustaining their acidic milieu (9). Recent studies underscore the host endolysosomal system’s role in the entry, replication, and egress of betacoronaviruses, including severe acute respiratory syndrome coronavirus 2 (SARS-CoV-2) and mouse hepatitis virus (MHV) (10, 11). ADP-ribosylation factor-like 8b (Arl8b)-mediated lysosomal transport to the cell periphery is particularly essential for viral egress (11). Using PHEV as a model, our recent study demonstrated that the virus traffics to lysosomes before undergoing Arl8b-dependent lysosomal exocytosis, an egress mechanism shared with SARS-CoV-2 and MHV (11, 12). Interestingly, while PHEV relies on V-ATPase-mediated lysosomal acidification for egress, SARS-CoV-2 and MHV utilize deacidified lysosomes (11, 12). However, the mechanisms underlying PHEV-induced lysosomal acidification remain poorly understood, prompting this investigation.

Building on this context, progranulin (PGRN), a lysosomal glycoprotein encoded by the granulin (*GRN*) gene, comprises 7.5 conserved granulin repeats (13, 14). PGRN traffics to lysosomes from the trans-Golgi network or extracellular space via two distinct pathways: (i) binding to sortilin, a VPS10 family transmembrane protein that acts as a high-affinity receptor for PGRN (15, 16), or (ii) interacting with prosaposin (PSAP), which binds to cation-independent mannose-6-phosphate receptor (CI-M6PR) independently of sortilin (17, 18). Within lysosomes, PGRN is cleaved into granulin peptides (GRNs), which perform intracellular and extracellular functions (13, 14). PGRN lysosomal targeting appears essential for its regulatory roles in lysosomal function (19). PGRN plays a critical role in maintaining lysosomal pH and degradative capacity, significantly impacting neuronal global proteostasis (20, 21).

Our previous studies revealed that PHEV release depends on lysosomal acidification and that PHEV infection reduces full-length PGRN expression (8, 12). However, the role of PGRN in PHEV-induced lysosomal acidification and viral release remains unclear. In this study, we demonstrate that PHEV infection decreases full-length PGRN levels while enhancing its lysosomal trafficking. PGRN knockout (KO) mice show resistance to PHEV infection, and PHEV enhances PGRN lysosomal targeting through sortilin- and CI-M6PR-mediated pathways. This targeting increases V-ATPase recruitment, elevating lysosomal acidity and promoting Arl8b-dependent lysosomal exocytosis. Additionally, *in vivo* neuronal PGRN knockdown, achieved through *in situ* injection of an adeno-associated virus (AAV)-mediated, neuron-specific short hairpin RNA (shRNA), inhibits PHEV transmission in mouse brain tissues. Collectively, these findings underscore the critical role of PGRN-mediated lysosomal acidity in driving lysosomal exocytosis-mediated PHEV release both *in vitro* and *in vivo*.

## RESULTS

### Links between PGRN and PHEV infection

**Fig 1:**
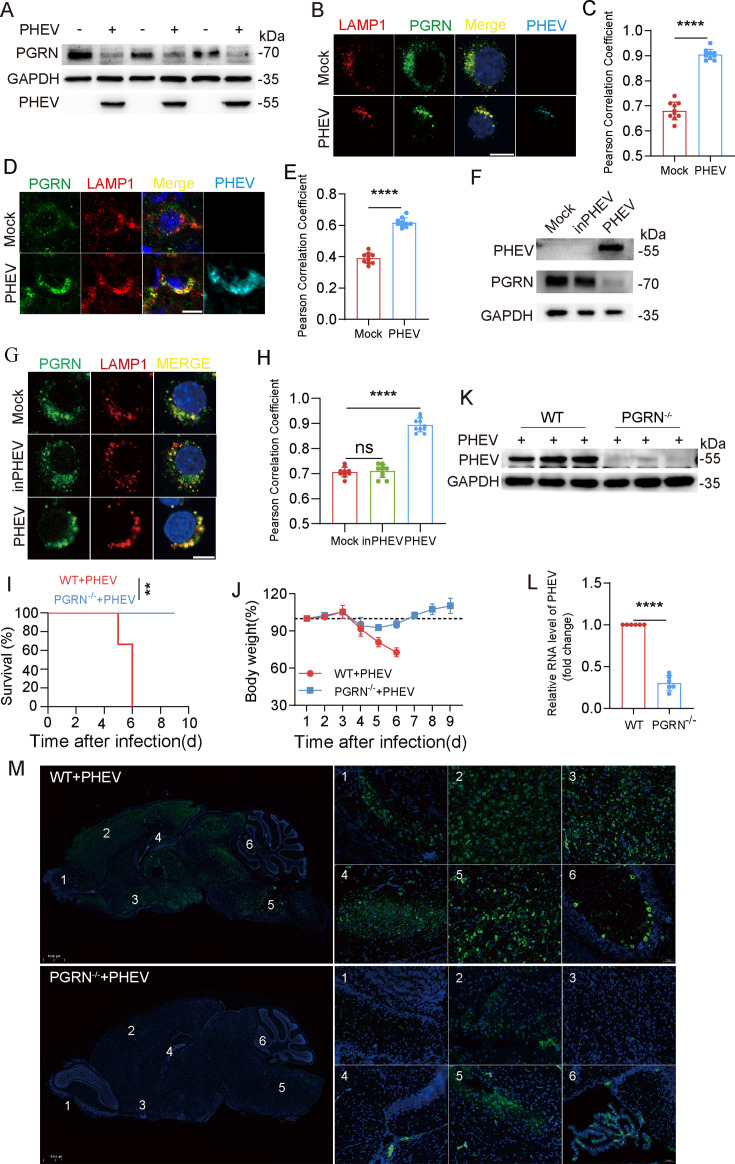
The links between PGRN and PHEV infection. (**A**) PGRN protein levels in PHEV- or mock-infected cells at 48 hpi were investigated by Western blotting analysis. Experiments were repeated three times. (**B**) Colocalization between LAMP1 and PGRN. Mock- or PHEV-infected cells were immunostained with anti-LAMP1 (red), anti-PGRN (green), and anti-PHEV (teal) antibodies. Scale bar: 10 µm. (**C**) The Pearson’s correlation coefficient was used to quantify the degree of colocalization between LAMP1 and PGRN in panel B (*n* = 10 cells examined over three independent experiments). (**D**) The brain tissue of mice intranasally inoculated with PHEV (10^6.80^ TCID_50_/0.1 mL) was immunostained with anti-LAMP1 (red), anti-PGRN (green), and anti-PHEV (teal) antibodies. Scale bar: 10 μm. (**E**) Pearson’s correlation coefficient was used to quantify the degree of colocalization between LAMP1 and PGRN in panel D (*n* = 10 cells examined over three independent experiments). (**F**) PHEV, PGRN, and GAPDH protein levels of mock-, inPHEV- or PHEV-infected N2a cells were investigated by Western blotting analysis, respectively. Experiments were repeated three times. (**G**) Colocalization of LAMP1 (red) with PGRN (green) in mock-, inPHEV-, or PHEV-infected N2a cells at 48 hpi. Scale bar: 10 μm. (**H**) Pearson’s correlation coefficient was used to quantify the degree of colocalization between LAMP1 and PGRN in panel G (*n* = 10 cells examined over three independent experiments). (**I**) Changes in body weights of mice in each group were monitored daily (*n* = 6). (**J**) Kaplan-Meier survival curve showing survival time of PHEV-infected wild-type (WT) or *Grn^-/-^* mice. (**K**) Western blotting analysis of PHEV nucleocapsid (N) and GAPDH protein in PHEV-infected WT or *Grn^-/-^* mice brain. (**L**) PHEV N genomic RNA levels in PHEV-infected WT or *Grn^-/-^* mice brain. (**M**) PHEV-infected WT or *Grn^-/-^* mice brain sections were immunostained with anti-PHEV (green) antibodies. The above experiments were repeated three times. Representative blots and images are shown. Data are shown as mean ± SD. *P*-values were considered significant when *P* < 0.05 and denoted as **, *P* < 0.01, ****, *P* < 0.0001, ns, not significant.

To investigate the effects of PHEV infection on PGRN expression and distribution, we first examined PGRN expression in PHEV-infected N2a cells. The full-length PGRN protein levels were significantly reduced in infected cells compared to mock-infected controls at 48 h post-infection (hpi) (Fig. 1A), consistent with previous findings (8). Additionally, we observed a significant increase in PGRN colocalization with lysosomal-associated membrane protein 1 (LAMP1) in both N2a cells and mouse brain tissues following PHEV infection (Fig. 1B through E). Notably, exposure of cells to inactivated PHEV (inPHEV) did not alter PGRN expression or PGRN lysosomal targeting compared to mock-infected cells (Fig. 1F through H), suggesting that these changes depend on active viral infection.

Additionally, to investigate lysosomal PGRN cleavage during PHEV infection, we analyzed full-length PGRN and GRNs in lysosomes isolated from mock-infected or PHEV-infected N2a cells. The results revealed a significant increase in full-length PGRN levels, but no change in GRN levels, within PHEV-hijacked lysosomes (Fig. S1A). These results indicate that PHEV infection enhanced PGRN lysosomal targeting. Furthermore, we observed increased levels of both full-length PGRN and GRNs in the supernatant of PHEV-infected N2a cells compared to mock-infected cells (Fig. S1B). The increase in extracellular full-length PGRN and GRNs likely results from PHEV-induced enhancement of lysosomal exocytosis. However, whether PHEV infection directly affects the processing of PGRN to GRNs remains unclear.

To further explore the role of PGRN in PHEV infection, we employed PHEV-permissive mice as an *in vivo* model system for studying PHEV-induced central nervous system (CNS) pathology and viral replication (7, 12, 22). Wild-type (WT) and PGRN knockout (*Grn^-/-^*) mice were intranasally infected with 50 µL PHEV (10^6.80^ TCID_50_/0.1 mL) (Fig. S2). Strikingly, all *Grn*^⁻/⁻^ mice survived, whereas the WT group exhibited 100% mortality by 6 days post-infection (dpi) (Fig. 1I). Both groups began losing weight at 4 dpi; however, WT mice continued losing weight until death, while *Grn^-/-^* mice experienced significantly less weight loss and began to gain weight at 4–5 dpi (Fig. 1J). Western blotting, qPCR, and immunofluorescence (IF) analyses of brains collected at 5 dpi revealed significantly reduced PHEV nucleocapsid (N) protein and RNA expression in *Grn^-/-^* mice, along with fewer PHEV-positive cells across brain regions such as the olfactory bulb, cerebral cortex, piriform cortex, hippocampus, brain stem, and cerebellum (Fig. 1K through M). These findings demonstrate that *Grn^-/-^* mice are resistant to PHEV infection.

Collectively, our results suggest that PHEV infection alters the full-length PGRN level and PGRN lysosomal targeting, while PGRN loss mitigates PHEV infection *in vivo*. These findings implicate PGRN in PHEV-induced lysosomal dysfunction and neural dissemination.

### PHEV facilitates sortilin- and CI-M6PR-mediated lysosomal trafficking of PGRN

**Fig 2:**
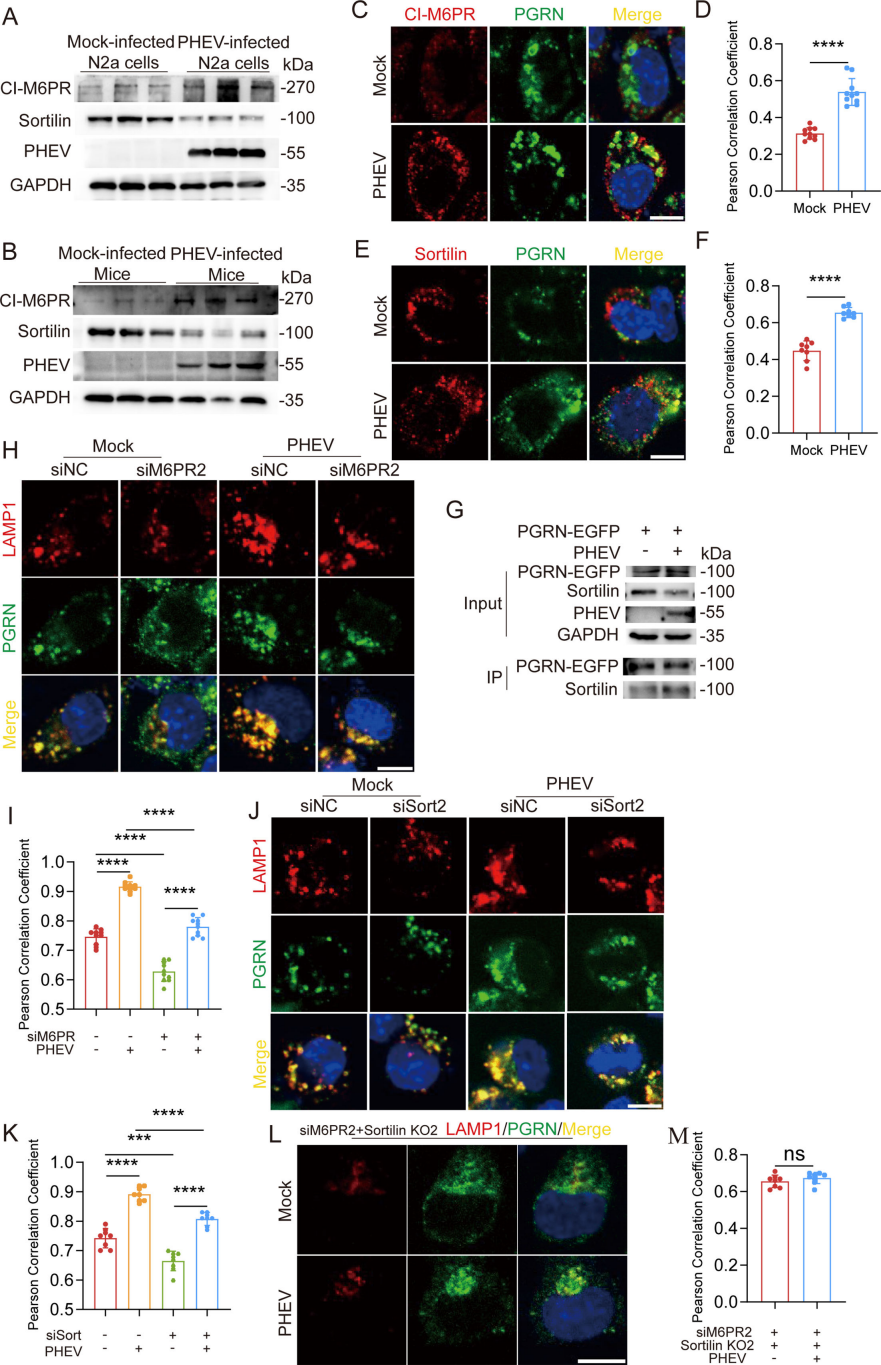
PHEV facilitates sortilin- and CI-M6PR-mediated lysosome trafficking of PGRN. (**A**) CI-M6PR, sortilin, PHEV N, and GAPDH protein level in mock- or PHEV-infected N2a cells, respectively. (**B**) CI-M6PR, sortilin, PHEV, and GAPDH protein level in mock- or PHEV-infected mice brain, respectively. (**C and E**) Colocalization of CI-M6PR/sortilin (red) with PGRN (green) in mock- or PHEV-infected N2a cells. Scale bar: 10 μm. (**D and F**) Pearson’s correlation coefficients of the images between CI-M6PR/sortilin and PGRN were analyzed using ImageJ (*n* = 10 or 8 cells). (**G**) Anti-GFP immunoprecipitates from PHEV- or mock-infected EGFP-PGRN-transfected N2a cells were harvested, and the physical interaction between PGRN and sortilin was investigated. (**H and J**) Colocalization of LAMP1 (red) with PGRN (green) in mock- or PHEV-infected siM6PR- and siSort-treated N2a cells, respectively. Scale bar: 10 μm. (**I and K**) Pearson’s correlation coefficients of the images between LAMP1 and PGRN were analyzed using ImageJ (*n* = 10 or 8 cells). (**L**) Colocalization of LAMP1 (red) with PGRN (green) in PHEV-infected siM6PR-treated sortilin KO cells. (**M**) Pearson’s correlation coefficients of the images between LAMP1 and PGRN were analyzed using ImageJ (*n* = 8 cells). The above experiments were repeated three times. Representative blots and images are shown. Data are shown as mean ± SD. *P*-values were considered significant when *P* < 0.05 and denoted as ****, *P* < 0.0001, ns, not significant.

PGRN lysosomal trafficking is regulated by key molecules, including the endocytic receptors sortilin and CI-M6PR (23). To investigate how PHEV infection affects these pathways, we examined sortilin and CI-M6PR expression *in vitro* using PHEV-infected N2a cells, and *in vivo* in mouse brain tissue. Western blot analysis revealed increased CI-M6PR expression and decreased sortilin expression in both models (Fig. 2A and B). IF assays further demonstrated that PHEV infection substantially enhanced PGRN colocalization with intracellular CI-M6PR and sortilin (Fig. 2C through F). Coimmunoprecipitation assays confirmed a strengthened physical interaction between PGRN and sortilin in PHEV-infected cells (Fig. 2G). These findings indicate that PHEV infection modulates sortilin and CI-M6PR expression while enhancing their interaction with PGRN.

To assess the contributions of these pathways to PHEV-induced PGRN lysosomal trafficking, we used small interfering RNAs (siRNAs) to knock down sortilin (siSort2) and CI-M6PR (siM6PR2) expression. Knockdown of either receptor significantly reduced PHEV-induced colocalization of PGRN and LAMP1, although complete elimination of expression was not achieved (Fig. S3A and B; Fig. 2H through K). Interestingly, even after knockdown, PGRN lysosomal targeting in PHEV-infected siSort2 or siM6PR2 cells remained significantly higher than in their mock-infected counterparts (Fig. 2H through K). Further analysis of sortilin knockout (KO2) cells treated with CI-M6PR siRNA revealed no significant difference in PGRN/LAMP1 colocalization between mock- and PHEV-infected cells (Fig. S3C; Fig. 2L and M). These results indicate that PHEV-induced lysosomal trafficking of PGRN depends on the concurrent participation of sortilin- and CI-M6PR-mediated pathways.

In addition, we found that PHEV N protein levels were significantly reduced in PHEV-infected siSort2 or siM6PR2 cells compared to PHEV-infected control cells. Further analysis of sortilin KO2 treated with CI-M6PR siRNAs revealed viral load was further decreased compared to knockdown of either sortilin or CI-M6PR (Fig. S4), indicating that sortilin- or CI-M6PR-mediated lysosomal trafficking of PGRN could play important roles in PHEV infection.

### PGRN promotes V-ATPase-dependent lysosomal acidification during PHEV infection

**Fig 3:**
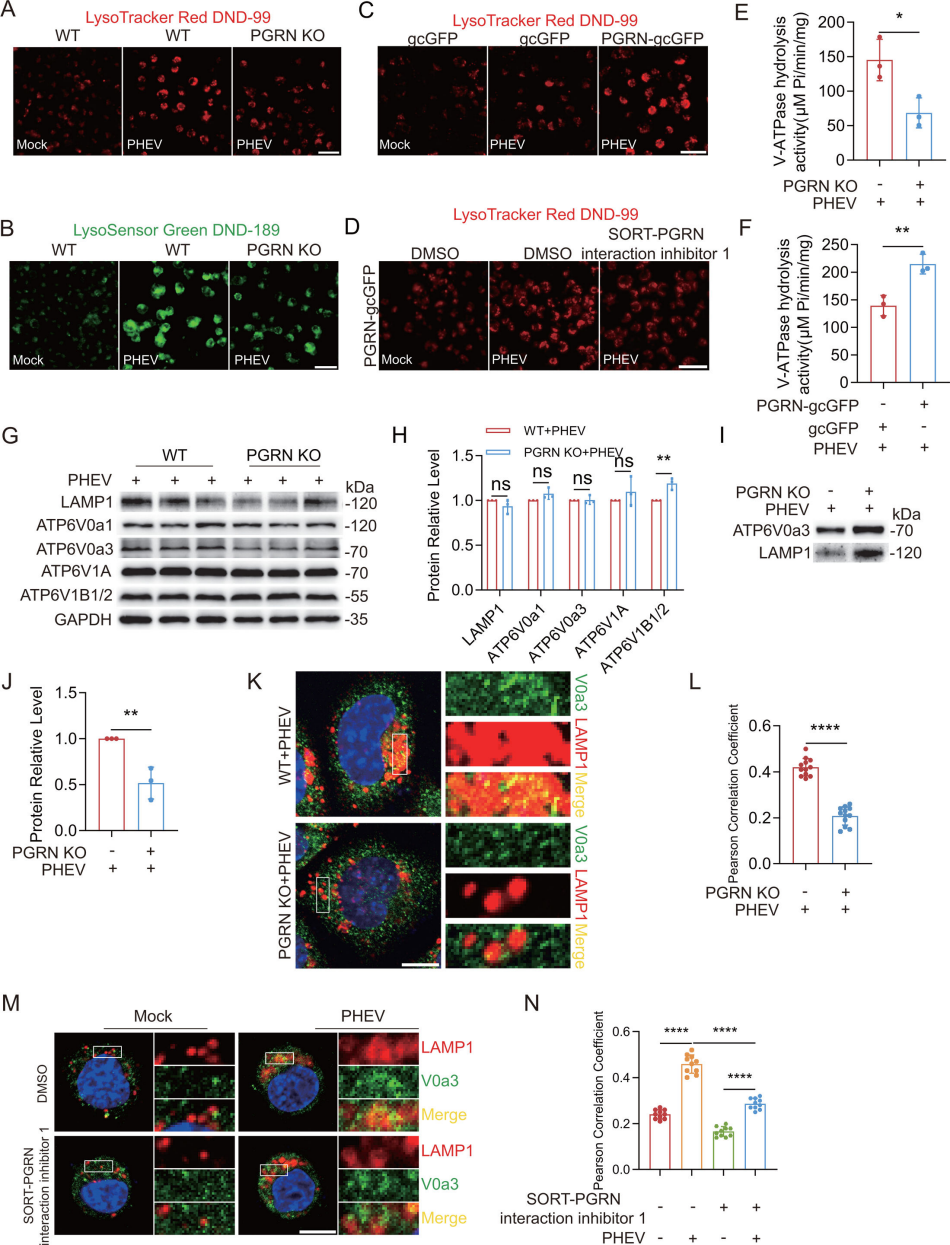
PGRN promotes V-ATPase-dependent lysosomal acidification during PHEV infection (**A**) PHEV-infected WT or PGRN KO N2a cells at 48 hpi were stained with LysoTracker Red DND-99. Scale bar: 40 μm. (**B**) PHEV-infected WT or PGRN KO N2a cells at 48 h were stained with LysoSensor Green DND-189. Scale bar: 40 μm. (**C**) PHEV-infected gcGFP or PGRN-overexpressing N2a cells at 48 hpi were stained with LysoTracker Red DND-99. Scale bar: 40 μm. (**D**) Mock- or PHEV-infected PGRN-overexpressing N2a cells with DMSO or SORT-PGRN interaction inhibitor 1 treatment were stained with LysoTracker Red DND-99. Scale bar: 40 μm. (**E**) The V-ATPase hydrolytic activity in the isolated lysosomes from PHEV-infected WT or PGRN KO cells at 48 hpi. (**F**) The V-ATPase hydrolytic activity in the isolated lysosomes from PHEV-infected PGRN-gcGFP or gcGFP overexpression cells at 48 hpi. (**G**) Western blot analysis of LAMP1, ATP6V0a1, ATP6V0a3, ATP6V1A, ATP6V1B1/2, and GAPDH from PHEV-infected WT or PGRN KO cells at 48 hpi, respectively. (**H**) LAMP1, ATP6V0a1, ATP6V0a3, ATP6V1A, and ATP6V1B1/2 protein levels in PHEV-infected WT or PGRN KO cells were quantified and normalized to GAPDH, respectively. (**I**) The protein levels of V0a3 in the isolated lysosomes from PHEV-infected WT and PGRN KO cells at 48 hpi, respectively. (**J**) The V0a3 protein levels in PHEV-infected WT and PGRN KO cells were quantified and normalized to LAMP1, respectively. (**K**) Colocalization of LAMP1 (red) with V0a3 (green) in PHEV-infected WT or PGRN KO cells. Scale bar: 10 μm. (**L**) The Pearson’s correlation coefficients of the images between V0a3 and LAMP1 were analyzed using ImageJ (*n* = 12 cells). (**M**) Colocalization of LAMP1 (red) with V0a3 (green) in mock- or PHEV-infected N2a cells with DMSO or SORT-PGRN interaction inhibitor 1 treatment. Scale bar: 10 μm. (**N**) Pearson’s correlation coefficients of the images between V0a3 and LAMP1 were analyzed using ImageJ (*n* = 10 cells). The above experiments were repeated three times. Representative blots and images are shown. Data are shown as mean ± SD. *P*-values were considered significant when *P* < 0.05 and denoted as *, *P* < 0.05, **, *P* < 0.01, ****, *P* < 0.0001, ns, not significant.

The pioneering study by Hasan et al., employing multimodal proteomics techniques, has provided preliminary evidence suggesting that PGRN plays a critical role in regulating lysosomal acidity (21). To investigate PGRN’s role in PHEV-induced lysosomal hyperacidification, we generated N2a monoclonal PGRN KO cell lines and PGRN-overexpressing cell lines using CRISPR/Cas9 or lentiviral vector systems, respectively (Fig. S5A and B). Lysosomal acidity was monitored in these cell lines after PHEV infection using LysoTracker Red DND-99 or LysoSensor Green DND-189 fluorescent acidotropic dyes. PGRN loss prevented PHEV-induced lysosomal acidification (Fig. 3A and B), while PGRN overexpression enhanced PHEV-induced lysosomal acidification (Fig. 3C).

Given that PHEV infection enhances PGRN lysosomal targeting, we investigated whether PGRN lysosomal trafficking influences PHEV-mediated lysosomal acidification. Treatment of PGRN-overexpressing cells with SORT-PGRN interaction inhibitor 1 for 24 h prior to 48 h exposure to infectious PHEV significantly reduced lysosomal acidification compared to DMSO-treated controls (Fig. S6A through C; Fig. 3D). These findings suggest that PGRN lysosomal trafficking positively regulates PHEV-induced lysosomal acidity.

Our earlier studies demonstrated that PHEV infection enhances lysosomal V-ATPase activity, which depends on the recruitment of the V0a3 subunit to lysosomal membranes (12). To explore the role of PGRN in V-ATPase activity during PHEV infection, we assessed V-ATPase ATP-hydrolytic activity in lysosomes isolated from PHEV-infected PGRN KO cells and PGRN-overexpressing N2a cells at 48 hpi. Remarkably, lysosomes from PHEV-infected PGRN KO cells exhibited significantly reduced ATP hydrolysis rates compared to WT cells, whereas lysosomes from PHEV-infected PGRN-overexpressing cells showed significantly increased activity compared to gcGFP-overexpressing controls (Fig. 3E and F). These results suggest that PGRN positively influences V-ATPase activity during PHEV infection.

Although our previous study showed that PHEV infection alters the expression of various V-ATPase subunits, including V0a1 and V0a3 (12), here, we found that PGRN KO had no effect on their expression levels in PHEV-infected cells (Fig. 3G and H). However, lysosomal fractions from PHEV-infected PGRN KO cells exhibited greatly diminished V0a3 protein levels compared to PHEV-infected WT cells (Fig. 3I and J). Additionally, PGRN loss significantly reduced V0a3 colocalization with LAMP1 in PHEV-infected PGRN KO cells compared to WT cells (Fig. 3K and L), indicating that PGRN enhances V0a3 recruitment to lysosomes.

To confirm the role of lysosomal PGRN in V0a3-dependent lysosomal acidification, cells were pretreated with SORT-PGRN interaction inhibitor 1 before PHEV infection. This treatment significantly reduced V0a3 and LAMP1 colocalization in PHEV-infected cells compared to untreated controls (Fig. 3M and N). These results underscore the pivotal role of PGRN lysosomal targeting in facilitating V0a3-dependent lysosomal acidification during PHEV infection.

### PGRN positively regulates PHEV-induced Arl8b-dependent lysosomal exocytosis

**Fig 4:**
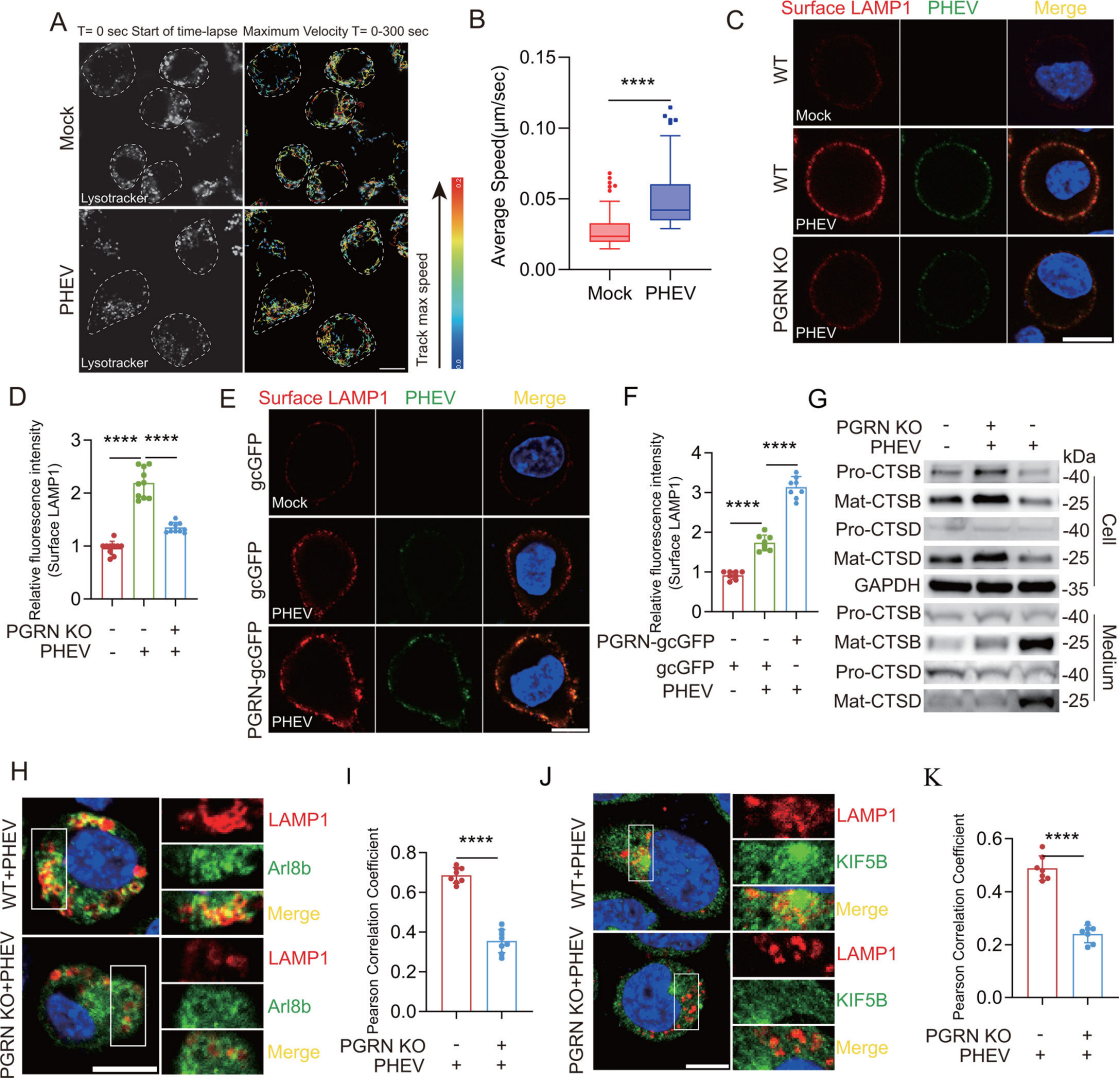
PGRN positively regulates PHEV-promoted Arl8b-dependent lysosomal exocytosis. (**A**) Mock- or PHEV-infected N2a cells were incubated with LysoTracker to label lysosomes. Left panels: representative confocal images of live N2a cells captured at the start of time-lapse imaging (T = 0 sec). Right panels: single-particle tracking analysis of LysoTracker-labeled lysosomes for T = 300 s with color-coding to show maximum velocity (blue, immobile; red, max mobility). Scale bar: 5 µm. (**B**) Tukey boxplot of the maximum average speed of LysoTracker-labeled lysosomes calculated from three independent live-cell imaging experiments. (**C**) Colocalization of surface LAMP1 (red) with PHEV (green) in PHEV-infected WT or PGRN KO cells at 48 hpi. Scale bar: 10 μm. (**D**) The surface LAMP1 levels on PHEV-infected WT or PGRN KO cells from the experiment whose results are shown in panel C were quantified using ImageJ (*n* = 10 cells). (**E**) Colocalization of surface LAMP1 (red) with PHEV (green) in PHEV-infected gcGFP- or PGRN-overexpressing cells at 48 hpi. Scale bar: 10 μm. (**F**) The surface LAMP1 levels on PHEV-infected gcGFP- or PGRN-overexpressing cells from the experiment whose results are shown in panel E were quantified using ImageJ (*n* = 8 cells). (**G**) The protein levels of Pro-CTSD, Pro-CTSB, Mat-CTSD, Mat-CTSB, and GAPDH at 48 hpi were analyzed by Western blotting, respectively. (**H**) Colocalization of LAMP1 (red) with Arl8b (green) in PHEV-infected WT or PGRN KO cells at 48 hpi. Scale bar: 10 μm. (**I**) Pearson’s correlation coefficients of the images between LAMP1 and Arl8b were analyzed using ImageJ (*n* = 8 cells). (**J**) Colocalization of LAMP1 (red) with KIF5B (green) in PHEV-infected WT or PGRN KO cells at 48 hpi. Scale bar: 10 μm. (**K**) Pearson’s correlation coefficients of the images between LAMP1 and KIF5B were analyzed using ImageJ (*n* = 7 cells). The above experiments were repeated three times. Representative blots and images are shown. Data are shown as mean ± SD. *P*-values were considered significant when *P* < 0.05 and denoted as ****, *P* < 0.0001.

Lysosomal exocytosis involves the migration of lysosomes to the cell periphery, where they fuse with the plasma membrane, releasing their luminal contents into the extracellular space (24, 25). This process requires lysosomes to travel along microtubules in an anterograde manner, facilitated by motor proteins such as kinesin (26, 27). However, the effects of PHEV infection on lysosomal motility remain unclear. Using LysoTracker dye to track lysosomal motility revealed that PHEV infection enhanced lysosomal movement (Fig. 4A and B). Additionally, PHEV infection increased anterograde lysosomal transport by recruiting kinesin motor proteins, as evidenced by the colocalization of LAMP1 and kinesin family member 5B (KIF5B) (Fig. S7).

We further investigated Arl8b, a small Arf-like GTPase in the Ras family, which localizes to late endosomes/lysosomes and facilitates their movement to the plasma membrane during exocytosis (11, 28). Our previous studies confirmed that PHEV infection enhances Arl8b-dependent lysosomal exocytosis (12). Here, we examined the relationship between PGRN and lysosomal exocytosis in PHEV-infected PGRN KO and PGRN-overexpressing cells. Cell surface staining for LAMP1 at 48 hpi revealed significantly lower plasma membrane LAMP1 (surface LAMP1) levels in PHEV-infected PGRN KO cells compared to WT cells (Fig. 4C and D). In contrast, surface LAMP1 levels were markedly increased in PHEV-infected PGRN-overexpressing cells compared to gcGFP-overexpressing cells (Fig. 4E and F).

We also measured extracellular levels of lysosomal proteases cathepsin D (CTSD) and cathepsin B (CTSB), along with their precursors (pro-CTSD/pro-CTSB) and respective mature forms generated through lysosomal proteolytic cleavage (Mat-CTSD/Mat-CTSB). PGRN KO cells exhibited significantly reduced extracellular release of Mat-CTSD and Mat-CTSB compared to WT cells (Fig. 4G), suggesting that PGRN positively influences PHEV-induced lysosomal exocytosis.

Further analysis revealed that PGRN depletion significantly reduced the colocalization of Arl8b and LAMP1 (Fig. 4H and I) as well as KIF5B and LAMP1 (Fig. 4J and K). This indicates that PGRN depletion impairs anterograde transport of PHEV-hijacked lysosomes by inhibiting Arl8b and kinesin recruitment to lysosomes. These results collectively suggest that PGRN promotes Arl8b-dependent lysosomal exocytosis during PHEV infection.

### PGRN-mediated lysosomal acidification positively regulates PHEV-enhanced lysosomal exocytosis

The role of lysosomal pH in regulating lysosomal exocytosis is debated, with reports suggesting that lysosomal alkalinization strongly stimulates lysosomal exocytosis in macrophages (29, 30). However, the effects of lysosomotropic drugs that enhance lysosomal acidification on lysosomal exocytosis remain unclear.

**Fig 5:**
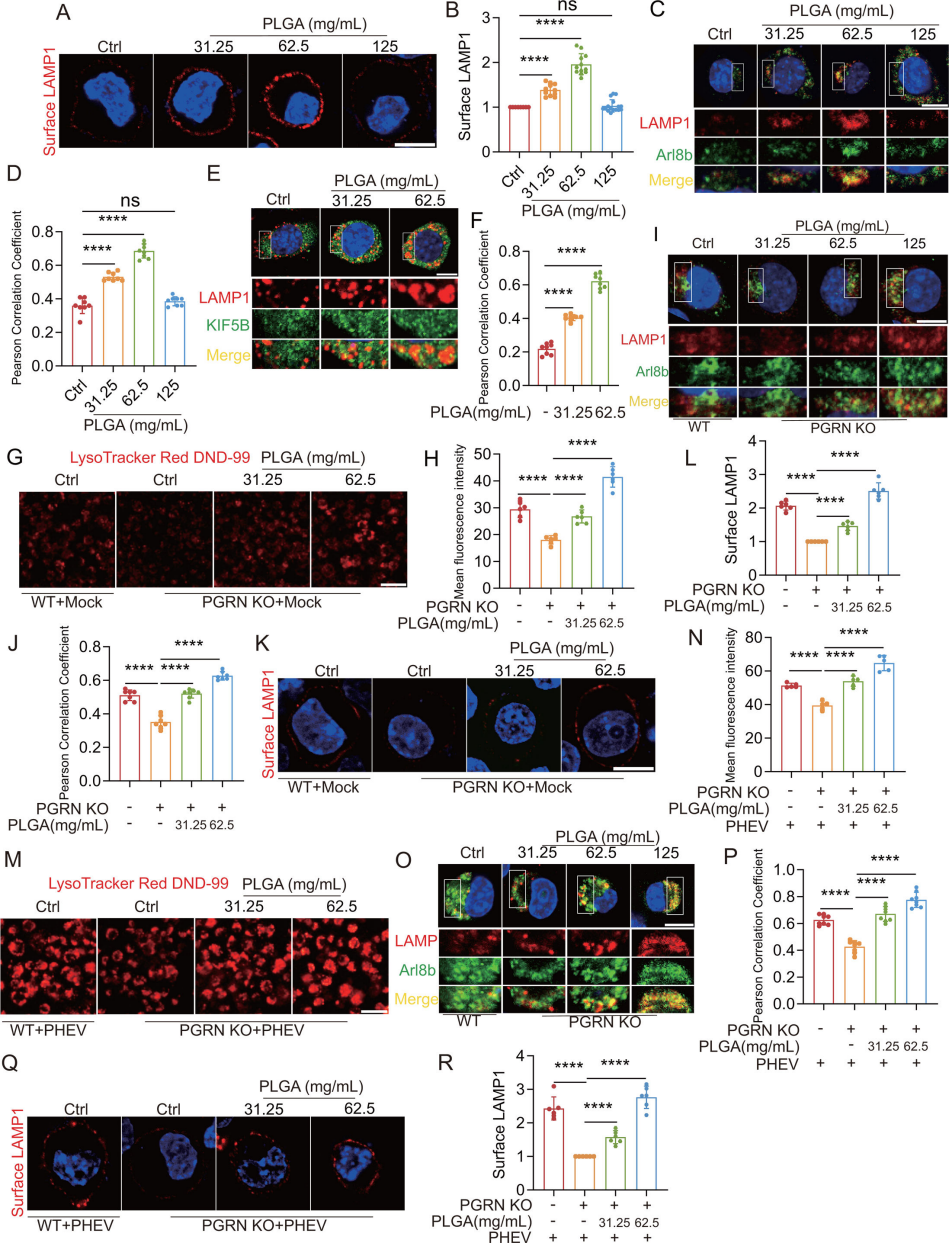
PGRN-mediated lysosomal acidification positively regulates PHEV-enhanced lysosomal exocytosis. (**A**) Surface LAMP1 (red) in N2a cells with different concentrations of PLGA for 24 h. Scale bar: 10 μm. (**B**) The surface LAMP1 levels in N2a cells from the experiment whose results are shown in panel C were quantified using ImageJ (*n* = 11 cells). (**C**) Colocalization of LAMP1 (red) with Arl8b (green) in different concentrations of PLGA-treated N2a cells. Scale bar: 10 μm. (**D**) Pearson’s correlation coefficients of the images between LAMP1 and Arl8b were analyzed using ImageJ (*n* = 8 cells). (**E**) Colocalization of LAMP1 (red) with KIF5B (green) in different concentrations of PLGA-treated N2a cells. Scale bar: 10 μm. (**F**) Pearson’s correlation coefficients of the images between LAMP1 and KIF5B were analyzed using ImageJ (*n* = 8 cells). (**G**) WT cells or PGRN KO cells with different concentrations of PLGA treatment for 24 h were stained with LysoTracker Red DND-99. Scale bar: 40 µm. (**H**) Quantification of LysoTracker Red DND-99 fluorescence intensity. (**I**) Colocalization of LAMP1 (red) with Arl8b (green) in different concentrations of PLGA-treated PGRN KO cells or WT cells. Scale bar: 10 µm. (**J**) The Pearson’s correlation coefficients of the images between LAMP1 and Arl8b were analyzed using ImageJ (*n* = 7 cells). (**K**) Colocalization of surface LAMP1 (red) with PHEV (green) in mock-infected WT cells or PGRN KO cells with different concentrations of PLGA treatment for 24 h. Scale bar: 10 µm. (**L**) The surface LAMP1 levels in N2a cells from the experiment whose results are shown in panel K were quantified using ImageJ (*n* = 6 cells). (**M**) PHEV-infected WT cells or PGRN KO cells with different concentrations of PLGA treatment for 24 h were stained with LysoTracker Red DND-99. Scale bar: 40 µm. (**N**) Quantification of LysoTracker Red DND-99 fluorescence intensity. (**O**) Colocalization of LAMP1 (red) with Arl8b (green) in different concentrations of PLGA-treated PHEV-infected PGRN KO cells. Scale bar: 10 µm. (**P**) The Pearson’s correlation coefficients of the images between LAMP1 and Arl8b were analyzed using ImageJ (*n* = 8 cells). The above experiments were repeated three times. (**Q**) Colocalization of surface LAMP1 (red) with PHEV (green) in PHEV-infected WT cells or PGRN KO cells with different concentrations of PLGA treatment for 24 h. Scale bar: 10 µm. (**R**) The surface LAMP1 levels in N2a cells from the experiment whose results are shown in panel Q were quantified using ImageJ (*n* = 6 cells). The above experiments were repeated three times. Representative blots and images are shown. Data are shown as mean ± SD. *P*-values were considered significant when *P* < 0.05 and denoted as ****, *P* < 0.0001, ns, not significant.

To explore this, we assessed the cell viability (via CCK8 assay) and lysosomal acidification (using LysoTracker fluorescence intensity) of N2a cells treated with varying concentrations of poly(lactic-co-glycolic acid) (PLGA; 31.25, 62.5, and 125 mg/mL), a biodegradable organic polymer internalized via endocytosis and metabolized within lysosomes (31). Lysosomal acidification gradually increased with increasing PLGA concentration, while cell viability remained unaffected (Fig. S8A through C). Interestingly, increasing PLGA concentrations from 31.25 to 62.5 mg/mL enhanced plasma membrane LAMP1 levels, but 125 mg/mL PLGA had no significant effect compared to untreated cells (Fig. 5A and B). Similarly, 31.25–62.5 mg/mL PLGA increased Arl8b/LAMP1 colocalization and Mat-CTSD/Mat-CTSB release compared to untreated cells, while 125 mg/mL had no effect (Fig. 5C and D; Fig. S9). Moreover, PLGA treatment from 31.25 to 62.5 mg/mL led to increased localization of KIF5B and LAMP1 (Fig. 5E and F). These results suggest that lysosomal acidification within a specific range (below 62.5 mg/mL PLGA) promotes lysosomal exocytosis by enhancing Arl8b and kinesin recruitment to lysosomes in N2a cells.

The relationship between PHEV-induced lysosomal acidification and exocytosis has remained unclear, despite evidence of both processes occurring simultaneously (12). Further analysis revealed that lysosomal acidity in PHEV-infected N2a cells at 48 hpi fell within the range induced by 31.25–62.5 mg/mL PLGA treatment (Fig. S10A through D). Given that lysosomal exocytosis is significantly enhanced during PHEV replication within this timeframe (12), our findings suggest that lysosomal acidification supports lysosomal exocytosis during PHEV infection.

The aforementioned observations highlight how PHEV-induced dysregulated PGRN expression and lysosomal trafficking can affect lysosomal acidification, emphasizing the significant role of PGRN in lysosomal exocytosis during PHEV replication. Nonetheless, further investigations are necessary to fully understand the relationship between PGRN-mediated lysosomal acidification and PHEV-enhanced lysosomal exocytosis. To further elucidate this relationship, we treated uninfected and PHEV-infected PGRN KO cells with PLGA (31.25 or 62.5 mg/mL) for 24 h and assessed lysosomal acidification (via LysoTracker fluorescence), Arl8b localization (via Arl8b/LAMP1 colocalization), and surface LAMP1 levels (via cell surface staining for LAMP1). PLGA treatment restored lysosomal pH, facilitated Arl8b lysosomal recruitment, and increased plasma membrane LAMP1 levels in both uninfected (Fig. 5G through L) and PHEV-infected PGRN KO cells (Fig. 5M through R). These results highlight the role of PGRN-mediated lysosomal acidification in promoting PHEV-induced lysosomal exocytosis.

### PGRN expression and lysosomal targeting enhance PHEV release

Building on our previous discoveries, we confirmed that PHEV egress depends on the lysosomal exocytosis pathway (12). Furthermore, our data corroborate the positive influence of PGRN-mediated lysosomal acidification on PHEV-induced lysosomal exocytosis, indicating pivotal roles of PGRN expression and lysosomal trafficking in PHEV release.

**Fig 6:**
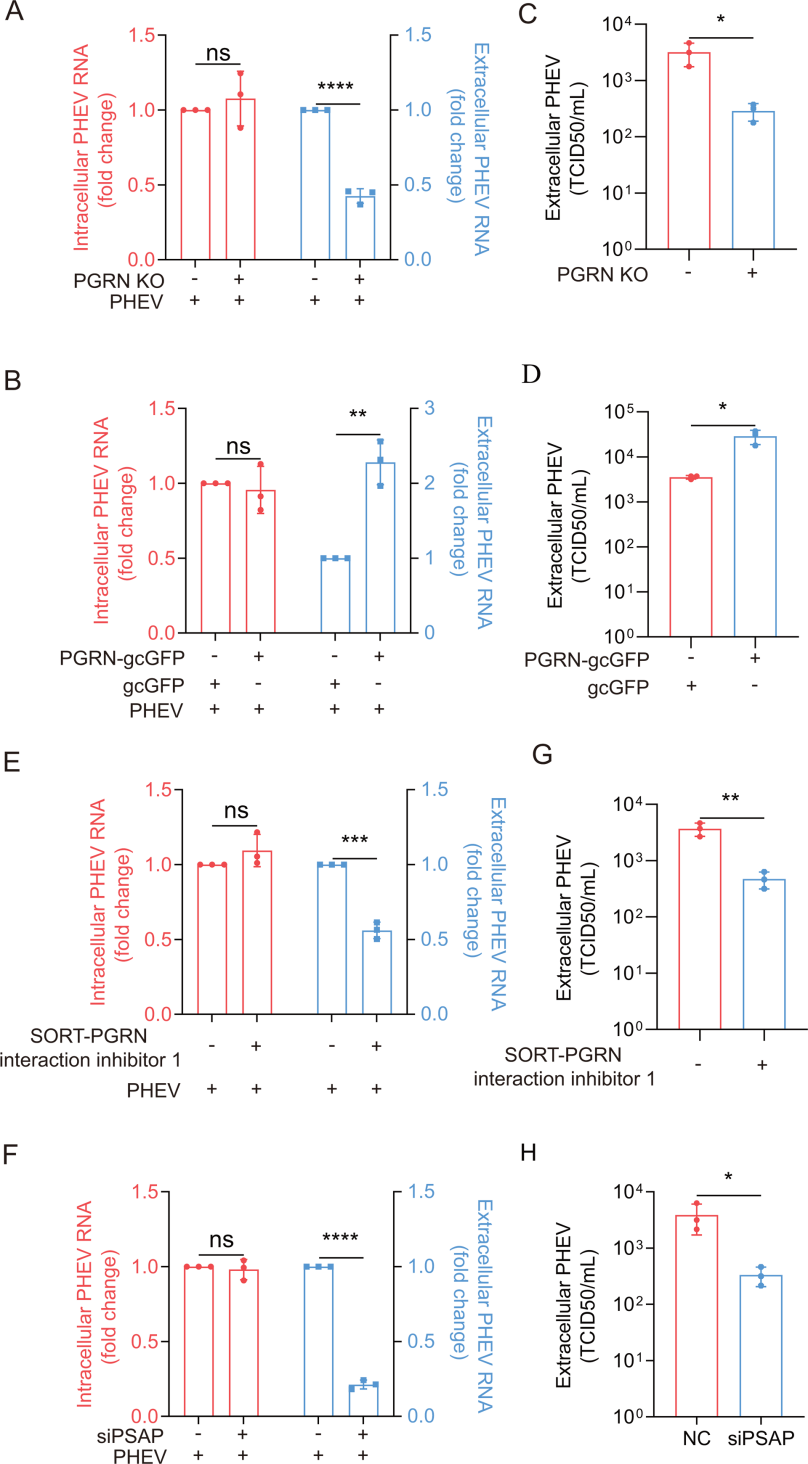
PGRN expression and lysosomal trafficking enhance PHEV release. (**A**) The PHEV N genomic RNA was determined using qPCR in PHEV-infected WT and PGRN KO cells at 48 hpi, respectively. (**B**) The PHEV N genomic RNA was determined using qPCR in gcGFP- and PGRN-gcGFP-overexpressing cells at 48 hpi, respectively. (**C and D**) Viral titers of culture supernatants in panels A and B by TCID_50_ endpoint assay. (**E and F**) The PHEV N genomic RNA was determined using qPCR in PHEV-infected SORT-PGRN interaction inhibitor 1 and siPSAP-treated cells at 48 hpi, respectively. (**G and H**) Viral titers of culture supernatants in panels C and D by TCID_50_ endpoint assay. The above experiments were repeated three times. Representative blots and images are shown. Data are shown as mean ± SD. *P*-values were considered significant when *P* < 0.05 and denoted as *, *P* < 0.05, **, *P* < 0.01, ***, *P* < 0.001, ****, *P* < 0.0001, ns, not significant.

To investigate the contribution of PGRN to PHEV egress, we quantified intracellular viral N genomes (via qPCR) and viral loads in culture supernatants (via TCID_50_ assay) in PGRN KO and PGRN-overexpressing cell lines. While PGRN expression had no significant impact on intracellular virus replication in either PHEV-infected PGRN KO or PGRN-overexpressing cells (Fig. 6A and B), PGRN deficiency significantly inhibited viral release (Fig. 6A and C). Conversely, PGRN overexpression enhanced PHEV release (Fig. 6B and D), highlighting the positive effect of PGRN on PHEV egress without influencing intracellular viral load in N2a cells.

Notably, viral release occurred without cell lysis under our experimental conditions, as evidenced by the absence of increased plasma membrane permeability, confirmed by trypan blue and propidium iodide exclusion assays (Fig. S11A and B). Consistent results were obtained under conditions of PGRN knockdown and transient PGRN-gcGFP overexpression (Fig. S12A through D), further validating the role of PGRN in enhancing viral release.

To explore the impact of PGRN lysosomal trafficking on PHEV release, following treatment with PSAP-targeting siRNA (siPSAP), we infected N2a cells with PHEV. Separately, we infected either N2a cells or PGRN-overexpressing cells with PHEV, in the presence or absence of the SORT1-PGRN interaction inhibitor 1 (Fig. S6A through C and S13A through C). Both treatments significantly impeded PHEV release without affecting intracellular viral loads (Fig. 6E through H; Fig. S14). Importantly, viral egress occurred without cell lysis (Fig. S11C and D), underscoring the crucial role of PGRN lysosomal trafficking in facilitating PHEV egress.

### PGRN regulates PHEV neural transmission in the CNS

PHEV, a neurotropic betacoronavirus, predominantly infects nerve cells over glial cells in the CNS, utilizing neural transmission for dissemination (32). Prior studies using PHEV-permissive mouse models of viral infection have highlighted the essential role of lysosomes in neural transmission of PHEV (12).

**Fig 7:**
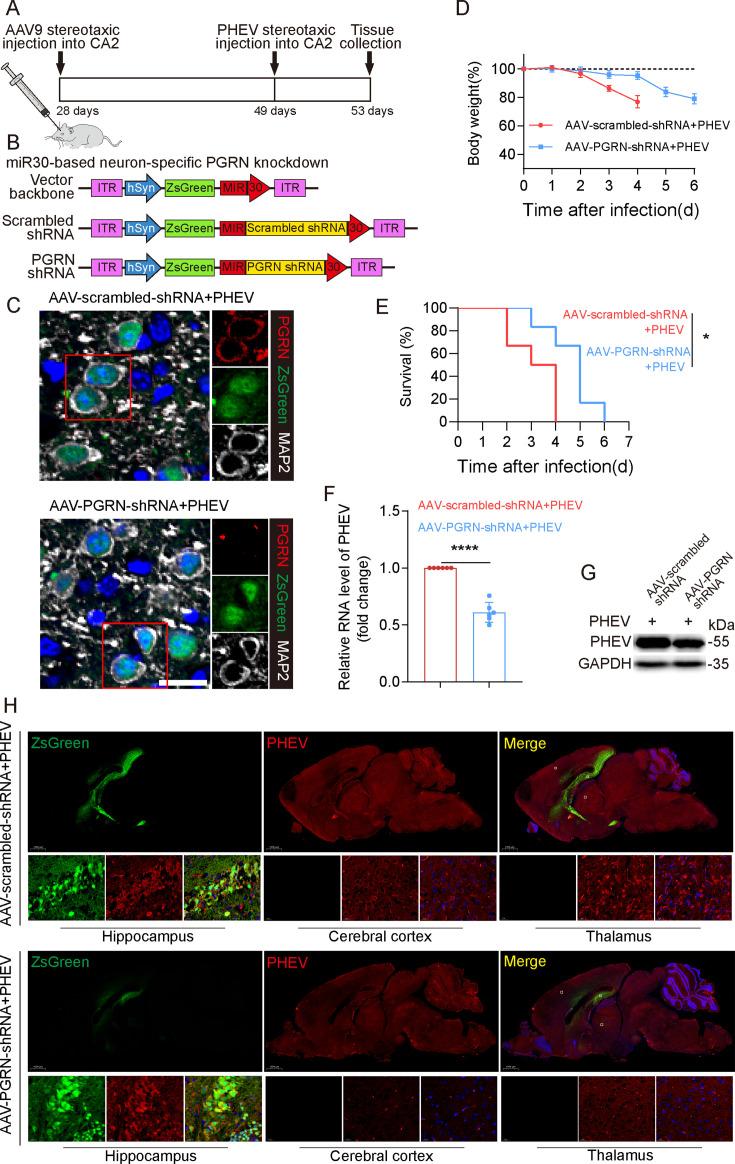
Knockdown inhibits PHEV neural transmission in CNS. (**A**) Schematic diagrams illustrating the experimental design for time-of-AAV treatment and sacrifice experiments. (**B**) AAV-scrambled shRNA or PGRN shRNA construct. (**C**) PHEV-infected AAV-scrambled shRNA- or AAV-PGRN shRNA-pretreated mice brain sections were immunostained with anti-PGRN (red) and anti-MAP2 (gray) antibodies. Representative images are shown. Scale bar: 20 µm. (**D**) Kaplan-Meier survival curve showing survival time of PHEV-infected mice pretreated with AAV-scrambled shRNA or AAV-PGRN shRNA (*n* = 6). (**E**) Changes in body weights of mice in each treatment group were monitored daily. (**F**) PHEV N genomic RNA levels in PHEV-infected AAV-scrambled shRNA- or AAV-PGRN shRNA-pretreated mice brain. (**G**) Western blotting analysis of PHEV N and GAPDH protein in PHEV-infected AAV-scrambled shRNA- or AAV-PGRN shRNA-pretreated mice brain. (**H**) PHEV-infected AAV-scrambled shRNA- or AAV-PGRN shRNA-pretreated mice brain sections were immunostained with anti-PHEV (red) antibodies. The above experiments were repeated three times. Representative blots and images are shown. Data are shown as mean ± SD. *P*-values were considered significant when *P* < 0.05 and denoted as *, *P* < 0.05; **, *P* < 0.01.

To further elucidate PGRN’s role in PHEV neural transmission, we employed AAV-mediated, neuron-specific delivery of a PGRN-targeting shRNA (AAV-PGRN-shRNA) to knock down PGRN expression, and a PGRN-overexpressing lentiviral vector (AAV-PGRN-3xFlag) to increase PGRN levels in mouse CNS neurons *in vivo*. Specifically, prior to PHEV infection, AAV-PGRN-shRNA or AAV-PGRN-3xFlag was delivered via *in situ* injection into the CA2 hippocampal region. Subsequently, mice received orthotopic PHEV injection at the same site, monitored daily for weight changes and symptoms over 21 days, followed by tissue collection 4 dpi (Fig. 7A and B; Fig. S15A).

Mice pretreated with AAV-PGRN-shRNA exhibited lower expression of PGRN in the CA2 hippocampal region, and higher survival rates and delayed onset of weight loss compared to those treated with AAV-scrambled-shRNA (Fig. 7C through E). Furthermore, AAV-PGRN-shRNA significantly reduced PHEV N protein and RNA levels in brain tissues (Fig. 7F and G). Notably, while hippocampus-specific PGRN knockdown did not affect viral loads in the hippocampal region, it significantly reduced viral loads in the cerebral cortex and thalamus (Fig. 7H), indicating a region-specific negative effect of PGRN knockdown on neural transmission that did not impact intracellular PHEV load at the site of initial PHEV infection. In contrast, PGRN overexpression induced by AAV-PGRN-3xFlag treatment did not affect survival rate, body weight, or PHEV transmission (Fig. S15B through F), suggesting that the weak effect of exogenous PGRN on PHEV neural transmission was attributed to sufficient endogenous PGRN levels enabling efficient transmission in mice. In summary, these findings underscore the pivotal role of PGRN in PHEV neural transmission within the CNS.

## DISCUSSION

Lysosomes, often referred to as cellular “garbage disposals,” play pivotal roles in nearly all eukaryotic cells and have been implicated in numerous studies supporting the replication of betacoronaviruses, including SARS-CoV-2, MHV, and PHEV (11, 12). These viruses use the Arl8b-dependent lysosomal exocytic pathway for egress, potentially contributing to lysosomal disorders. However, the mechanisms governing egress vary across betacoronaviruses, with SARS-CoV-2 and MHV utilizing deacidified lysosomes, while PHEV relies on V-ATPase-dependent lysosomal acidification for release (11, 12). These differing strategies highlight the virus-specific adaptations of lysosomal hijacking, warranting further investigation to uncover therapeutic targets and understand virus-host interactions.

The glycoprotein PGRN, which has been implicated in neurodegenerative diseases such as frontotemporal dementia, lysosomal storage disorders, and neuronal ceroid lipofuscinosis, traffics to lysosomes, where it is processed to GRNs and performs critical functions, including regulating lysosomal pH and degrading proteins and lipids (19, 20). Current research indicates that PGRN exhibits a dynamic distribution across cellular compartments, with primary localization within the secretory pathway and lysosomal system (23). Synthesized in the rough endoplasmic reticulum and post-translationally modified in the Golgi apparatus, PGRN is constitutively secreted into the extracellular space via exocytosis (33, 34). We found that PHEV infection significantly decreases full-length PGRN levels throughout the cell. During PHEV infection, lysosomes in nerve cells show increased levels of full-length PGRN but unchanged GRN levels, suggesting enhanced lysosomal targeting of PGRN. Furthermore, both full-length PGRN and GRN levels increase in the supernatant of PHEV-infected N2a cells compared to mock-infected cells, likely due to PHEV-triggered lysosomal exocytosis. It remains unclear if PHEV directly affects the conversion of PGRN to GRNs.

Importantly, the PHEV-mediated decrease in cellular full-length PGRN levels is not solely attributable to increased PGRN lysosomal cleavage but also involves reduced *GRN* mRNA expression at specific replication stages (8). Notably, during early PHEV replication stages, PGRN protein levels decreased significantly despite stable *GRN* mRNA expression (data not shown). These results indicate that the mechanism underlying PHEV-decreased full-length PGRN levels involves complex regulatory pathways. Whether PHEV infection impacts PGRN synthesis and post-translational modification warrants further study.

Additionally, sortilin and CI-M6PR, as multifunctional receptors, play multiple roles in numerous pathophysiological processes (35, 36). We observed that PHEV infection significantly influenced PGRN lysosomal trafficking via sortilin- and CI-M6PR-mediated pathways. PHEV infection enhanced PGRN lysosomal targeting through regulation of receptor expression, receptor lysosomal recruitment, and receptor-protein interactions. PHEV load was significantly reduced after knocking down sortilin or CI-M6PR in N2a cells, indicating that these multifunctional receptors likely play important roles in PHEV infection.

Lysosomal pH is maintained primarily by V-ATPase, a pump that transports protons across the lysosomal membrane (9, 37). V-ATPase comprises two functionally distinct domains, V0 (transmembrane) and V1 (cytosolic), which dynamically assemble to form the active proton pump (9). During PHEV replication, PGRN positively regulated lysosomal acidification by enhancing the lysosomal distribution of V0a3, a key V-ATPase subunit, while modulating the expression of V1 subunits B1 and B2. Interestingly, our results suggest that PGRN loss attenuated PHEV-induced lysosomal acidification by restoring PHEV-reduced V1 subunit expression while limiting V0a3 lysosomal recruitment. These findings underscore the intricate role of PGRN in modulating V-ATPase activity and lysosomal acidification during PHEV infection (12). Notably, Hasan et al. found that the loss of PGRN led to an elevation of lysosomal pH, accompanied by increased levels of various V-ATPase subunits within the cytosolic V1 domain, such as V1 subunit B1 and B2 (21). This alteration potentially disrupts the attachment of the V1 domain to the stationary membrane V0 domain, a crucial step required for efficient proton transport across the lysosomal membrane (21). Intriguingly, our findings revealed that PGRN loss attenuated the PHEV-enhanced V0a3 lysosomal distribution while simultaneously restoring the reduced expression of V1 subunits B1 and B2 caused by PHEV infection (Fig. 3G and H) (12). These results suggest that PGRN loss modulates V-ATPase activity by regulating the localization and expression of its subunits in both V1 and V0 domains, thereby mitigating PHEV-induced lysosomal acidification. However, the relationship between PGRN and lysosomal acidification is intricate and multifaceted. Our understanding of how PGRN expression and lysosomal trafficking regulate V-ATPase activity (specifically through influencing subunit expression and distribution) remains incomplete. Further exploration of the structural dynamics of V-ATPase in lysosomes with aberrant expression is necessary. Advanced techniques, like electron cryo-microscopy, could offer key insights into this complex interplay and unravel its nuances.

Lysosomes, dynamic organelles, traverse the cell to the plasma membrane in preparation for exocytosis (30). This process is orchestrated by a network of regulatory proteins, including the BLOC-one-related complex, the small GTPase Arl8b, and the adaptor protein PLEKHM2 (also known as SKIP), which collectively enable lysosome coupling to kinesins for microtubule-based transport (27). Previous studies noted variations in lysosomal pH by subcellular localization, with perinuclear lysosomes being more acidic than the alkaline peripheral lysosomes near the plasma membrane (29). This negative correlation between lysosomal acidity and exocytosis has been supported by findings that lysosomal alkalinization stimulates lysosomal exocytosis in macrophages (38). However, recent research by Ponsford et al. challenges this notion, reporting no significant difference in pH between perinuclear and peripheral lysosomes across various cell types (39). In this context, we explored the impact of lysosomal acidification on exocytosis in nerve cells. Modest lysosomal acidification induced by PLGA treatment enhances exocytosis by recruiting Arl8b- and kinesin-associated proteins. These findings suggest that lysosomal acidification within a specific pH range may augment exocytosis, but the relationship may vary across different cell types. Further investigation is warranted to delineate the precise effects of lysosomal acidity alterations on exocytosis and the function of Arl8b- and kinesin-associated ensembles in nerve cells.

PHEV, a neurotropic betacoronavirus, primarily infects neurons rather than glial cells in the CNS and utilizes neural transmission pathways for spread (32). We have demonstrated that PGRN facilitates PHEV egress from N2a cells without altering intracellular viral load. Given PHEV’s neurotropic nature and its pathogenic impact on the central nervous system, particularly damage to the hippocampus and cerebral cortex (7), we conducted a key experiment. Targeted knockdown of PGRN in hippocampal neurons resulted in a significant reduction in viral load in the cortex and thalamus, while viral accumulation within the hippocampus remained unchanged under our experimental conditions. This suggests that neuronal PGRN knockdown impedes interneuronal transmission of PHEV without affecting intracellular viral replication.

Previous studies have shown that PHEV undergoes retrograde transport within vesicular structures along microtubules toward the neuronal cell body (40). Furthermore, Li et al. systematically investigated the assembly and dissemination of PHEV (strain HEV 67N) within the primary motor cortex of infected rats. Their work demonstrated that the virus employs a membranous-coating-mediated mechanism of endo-/exocytosis for transsynaptic transfer, a process also capable of transporting larger particulate cargo like virions (40). Our previous work revealed that lysosomes play a critical role in PHEV neural transmission and virus-induced neuropathology in the CNS (12). Thus, newly assembled virions may be released via fusion of PHEV-hijacked lysosomes with the plasma membrane, followed by uptake into subsequent neurons via membranous-coating-mediated endocytosis.

We also observed a marked increase in lysosomal PGRN levels in mouse brain tissues following PHEV infection (Fig. 1D and E). Based on our *in vitro* data, we propose that during membranous-coating-mediated exocytosis for transsynaptic viral transfer, PGRN knockdown may attenuate V-ATPase activity, impair lysosomal acidification, and disrupt lysosomal exocytosis, potentially through compromised recruitment of KIF5B and Arl8b, thereby hindering PHEV spread within the CNS. Herein, we propose a model consistent with both *in vitro* and *in vivo* observations: PGRN serves as a critical host factor that enables efficient transsynaptic dissemination of PHEV. However, the precise mechanisms underlying PGRN-mediated neurotransmission of PHEV will require further validation through additional *in vivo* experimental studies.

**Fig 8:**
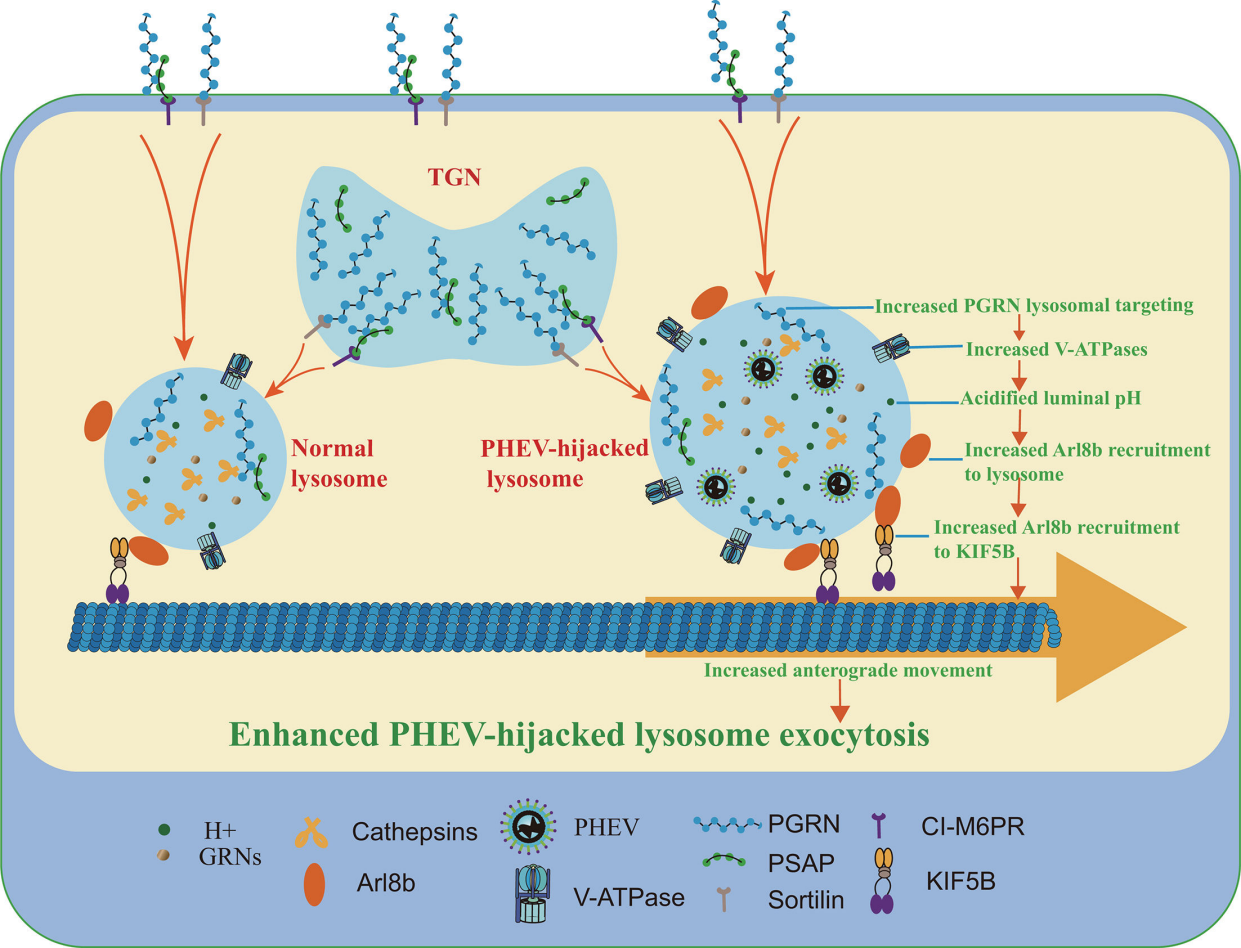
PGRN-mediated lysosomal acidity facilitates PHEV-hijacked lysosome exocytosis. PHEV infection results in enhanced PGRN lysosomal trafficking by facilitating sortilin- and CI-M6PR-mediated lysosomal trafficking of PGRN. Increased PGRN lysosomal trafficking caused by PHEV infection can elevate lysosomal recruitment of V-ATPase and further result in increased lysosomal acidity. This process triggers Arl8b-dependent lysosomal exocytosis, facilitating PHEV-hijacked lysosome exocytosis.

Previously, we found that PHEV localizes to lysosomes, inducing their enlargement (8, 12). This study extends those findings, showing that PGRN-mediated lysosomal acidification positively impacts PHEV release through an Arl8b-dependent lysosomal exocytic pathway, particularly in nerve cells. These insights highlight the intricate relationship between PGRN-mediated lysosomal acidification and PHEV-enhanced lysosomal exocytosis (Fig. 8). Moreover, emerging studies have revealed additional roles for PGRN in lysosomal biology, including its involvement in glucocerebrosidase activity, cathepsin activation, lipid degradation, and lysosomal membrane integrity regulation (19). These multifaceted functions suggest that PGRN enhances PHEV release through mechanisms beyond its effects on lysosomal acidity alone. Notably, our results indicate that increased lysosomal acidification induced by PLGA treatment could not fully compensate for the decrease in PHEV release caused by PGRN loss (data not shown), underscoring the multifaceted nature of PGRN’s impact on viral egress.

In conclusion, elucidating the intricate interplay between PGRN-mediated lysosomal acidification and viral egress provides a foundation for future therapeutic interventions against PHEV infection. Further research into this complex relationship will advance our understanding of lysosomal biology and its exploitation by pathogens.

## MATERIALS AND METHODS

### Mouse strains

*Grn^-/-^* mice were kindly provided by Dr. Jinhua Liu (College of Veterinary Medicine, China Agricultural University), Dr. Fanhua Wei (College of Agriculture, Ningxia University), and Dr. Xiaojing Ma (Department of Microbiology and Immunology, Weill Cornell Medical College). Genotypes were confirmed by sequencing PCR products generated from genomic DNA, as described previously (41). Western blot analysis of tissue lysates validated the absence of PGRN protein products.

### Antibodies and reagents

Primary antibodies used in this study included anti-Cathepsin D (ab75852), anti-Cathepsin B (ab214428), anti-sortilin (ab188586), anti-ATP6V1A (ab199326), and anti-ATP6V1B1 + ATP6V1B2 (ab200839) from Abcam; anti-PGRN (AF2557) from R&D Systems; anti-CD107a (553792) from BD Biosciences; anti-Arl8b (13049-1-AP), anti-ATP6V0a3 (12649-1-AP), anti-PSAP (10801-1-AP), anti-GFP (50430-2-AP), anti-CI-M6PR (20253-1-AP), anti-KIF5B (21632-1-AP), and anti-GAPDH (60004-1-Ig) from Proteintech; anti-ATP6V0a1 (sc-374475) from Santa Cruz Biotechnology; and anti-MAP2 (4542S) from Cell Signaling Technology.

Secondary antibodies included the following: HRP-conjugated goat anti-mouse IgG (SA00001-1), HRP-conjugated goat anti-rabbit IgG (SA00001-2), HRP-conjugated goat anti-rat IgG (SA00001-15), and HRP-conjugated goat anti-sheep IgG (SA00001-16) from Proteintech; anti-mouse Alexa 488 (4408), anti-mouse Alexa 647 (4410), anti-rabbit Alexa 488 (4412), anti-rabbit Alexa 647 (4414), and anti-rabbit Alexa 594 (8889) from Cell Signaling Technology; and anti-sheep Alexa 488 (ab150177) and anti-rat Alexa 594 (ab150160) from Abcam.

Other reagents included LysoTracker Red DND-99 (L7528) and LysoSensor Green DND-189 (L7535) from Thermo Fisher Scientific; Hoechst 33342 (C1022) and propidium iodide (ST511) from Beyotime; SORT-PGRN interaction inhibitor 1 (HY-115213) and PLGA (HY-B2247A) from MCE.

### Cell culture and viruses

N2a cells (ATCC) were cultured in DMEM medium (Gibco), supplemented with 10% fetal bovine serum (FBS) (Life Technologies, 10437) and 1% penicillin/streptomycin (Life Technologies, 15140), and maintained at 37°C and 5% CO_2_.

The PHEV strain used in this study, PHEV CC14 (GenBank accession number MF083115.1), was maintained in our laboratory. Cells were infected with PHEV at a concentration of 10^6.80^ TCID_50_/0.1 mL in DMEM containing 2% FBS for 1 h. Following the absorption period, cells were incubated in complete DMEM at 37°C in 5% CO_2_ for the designated experimental durations. Inactivated PHEV was prepared as previously described (42).

### Lentivirus production and infection

The lentivirus shuttle vector pLenti-GIII-CMV-PGRN-gcGFP and the control vector were constructed using standard molecular biology methods, as previously described (43). HEK293T cells were transfected with the lentivirus shuttle plasmid and auxiliary packaging plasmids pMD2.G and psPAX2. Virus-containing supernatants were collected over 72 h and centrifuged at 11,000 × *g* for 10 min to remove debris. The resulting virus-containing medium was used to infect N2a cells. Stable overexpression of PGRN-gcGFP or the control was confirmed by Western blotting after puromycin selection.

### Stereotaxic surgery and virus injection

The following vectors were used: AAV2/9-hSyn-ZsGreen-scrambled-shRNA (1.7 × 10^12^ v.g./mL), AAV2/9-hSyn-ZsGreen-PGRN -shRNA (1.8 × 10^12^ v.g./mL), AAV2/9-hSyn-ZsGreen-3×Flag (1.9 × 10^12^ v.g./mL), and AAV2/9-hSyn-ZsGreen-PGRN-3×Flag (1.8 × 10^12^ v.g./mL). All vectors were purchased from Hanheng Biotechnology and stored at −80°C.

Four-week-old BALB/c mice (Laboratory Animal Center of Jilin University) were deeply anesthetized with pentobarbital sodium and secured in a stereotaxic apparatus. AAV was unilaterally injected into the CA2 hippocampal region at a flow rate of 0.1 µL/min (total volume 5 µL). The pipette remained at the injection site for approximately 10 min post-injection to minimize tissue damage before being slowly retracted. Mice were orthotopically injected with 5 µL PHEV (10^6.80^ TCID_50_/0.1 mL) 21 days after AAV administration. At 5 dpi, mice were sacrificed by CO_2_ asphyxiation in accordance with animal handling guidelines. Brains were excised and placed on an ice pad for further testing, as described previously (12).

### RNA isolation and real-time qPCR (RT-qPCR)

Total RNA was extracted from cells using TRIzol reagent (TaKaRa Biotechnology) following the manufacturer’s protocol. Total RNA was reverse transcribed using All-In-One RT MasterMix (Abm). RT-qPCR was performed using 2× SYBR qPCR master mix (Biomake) with a QuantStudio 1 instrument (Thermo Fisher Scientific). PHEV RNA was detected as described previously using primer and probe sequences targeting the N gene of PHEV (44). A LightCycler 480 (Roche Applied Science) was used for qPCR. Thermocycling amplification conditions were as follows: 50°C for 30 min, 94°C for 15 min, followed by 45 cycles of (95°C for 15 s, 58°C for 60 s, and 72°C for 10 s), as previously described (44). GAPDH transcription levels served as the internal control.

### RNA interference

For RNA interference assays, siRNA oligos targeting sortilin (NM_001271599), CI-M6PR (NM_010515), and PSAP (NM_001146120) were purchased from GenePharma and prepared according to the manufacturer’s instructions. The sequences were as follows: Negative control: 5′-UUCUCCGAACGAGUCACGUTT-3′; siSort1: 5′-GCCCUUAAUGGGUCAGAATT-3′; siSort2: 5′-GCAAUGCCCAUGCUGUAUATT-3′; siM6PR1: 5′-CGGAGGAAAUACUACCUCAAUTT-3′; siM6PR2: 5′-CCUGCAGUGACAGUAACAUUUTT-3′; siPSAP1: 5′-GCAAGAAACUGGUCCUCUAUUTT-3′; siPSAP2: 5′-CCUGGAUUUGUGUGCUCGAAATT-3′; siPGRN1: 5′-GCCAACUACAGCUGCUGUATT-3′; siPGRN2: 5′-CCUGCUGUCCCUACCUAAATT-3′; siPGRN3: 5′-CCAACAGGGCAGUGUCUUUTT-3′.

N2a cells were transfected with the siRNA oligos using the GP-transfect-mate transfection reagent (GenePharma) according to the manufacturer’s instructions. Subsequent experiments were conducted 24 h post-transfection.

### Western blotting

Western blotting analysis was conducted as previously described (8). Briefly, brain tissue or N2a cells were lysed in RIPA buffer and centrifuged at 12,000 × *g* for 10 min. The supernatant was mixed with SDS loading buffer and boiled for 10 min. Proteins were separated by SDS-PAGE, transferred onto polyvinylidene difluoride membranes (Millipore), and probed with appropriate antibodies. Protein band densities were quantified and analyzed using Fiji software.

### Coimmunoprecipitation

N2a cells were transiently transfected with the PGRN-EGFP vector using Lipo8000 Transfection Reagent (Beyotime, C0533). After 24 h, cells were infected with PHEV (10^6.80^ TCID_50_/0.1 mL) for 48 h. Cell lysates were collected in IP buffer (50 mM Tris pH 8.0, 150 mM NaCl, 1% Triton X-100, 0.1% deoxycholate, and protease inhibitors) as described previously (45). Lysates (3 mg/mL) were precleared by rotating them overnight with 6 µg GFP antibody at 4°C and then incubated with 25 µL rProtein A/G Beads 4FF (Smart-Lifesciences, SA032100) for 1 h at 4°C with gentle agitation. After washing three times with wash buffer (BBI, C600482-0500), beads were heated at 95°C for 10 min in 1× SDS PAGE Protein Sampling Buffer (Servicebio, G2075) and used for Western blotting.

### Immunocytochemistry

Cells grown on glass coverslips were fixed with 4% paraformaldehyde at room temperature for 15 min and permeabilized with 0.1% Triton X-100 for 10 min. Blocking was performed with phosphate-buffered saline (PBS) containing 5% skim milk for 1 h at 37°C. Cells were incubated with primary antibodies overnight at 4°C, washed, and treated with Alexa Fluor-conjugated secondary antibodies (Alexa-488, Alexa-594, or Alexa-647, 1:400) for 1 h at room temperature. Coverslips were mounted with anti-fluorescence quenching sealant (Beyotime) after Hoechst staining. For surface LAMP1 staining, cells were processed as described previously (12) and imaged using an Olympus FV3000 confocal microscope.

### Establishment of knockout cell lines using CRISPR/Cas9

Knockout of PGRN and sortilin was performed using the CRISPR/Cas9 system. Single-guide RNAs targeting GRN (NM_008175.5, 5′-TGAGCGCCTTCCAGAGACGC-3′) and sortilin (5′-AAACAGGCCGTCCGCAGCTCCCCGC-3′) were cloned into the PX459 vector. Plasmids were transfected into N2a cells with Lipofectamine 3000. Stable cell lines were generated under puromycin selection (4 µg/mL) for 6 days, and gene knockout was confirmed by Western blot analysis.

### Measurement of V-ATPase activity

ATP hydrolysis was measured following a modified procedure by Lee et al. (46). Cells were processed as described previously (12), and measurements were conducted with and without the V-ATPase inhibitor bafilomycin A1 (1 µM). Absorbance was recorded at 750 nm, and KH_2_PO_4_ was used to generate the standard curve.

### LysoTracker Red and LysoSensor Green staining

Lysosomal acidity in PHEV- or mock-infected N2a cells was assessed using LysoTracker Red DND-99 (100 nM) or LysoSensor Green DND-189 (100 nM) for 1 h at 37°C. Cells were rinsed twice with warm PBS and imaged with a fluorescence microscope. Average fluorescence intensity per puncta area was quantified as described previously (12).

### Live-cell imaging

Mock- or PHEV-infected N2a cells were seeded on glass-bottom tissue culture-treated cell imaging dishes (Beyotime) and incubated with phenol red-free DMEM containing 100 nM LysoTracker Red DND-99 for 30 min at 37°C. Time-lapse imaging captured lysosome movements every 10 s for 5 min to create a video with 10 frames per second. Average lysosome speeds were analyzed using the TrackMate plugin in Fiji software, as described previously (47).

### Lysosomal protein isolation

Lysosomal proteins were extracted from cells using a lysosomal protein extraction kit (Beibo Biological Technology), following the manufacturer’s instructions. Briefly, the cells were harvested and washed with 1× PBS, followed by incubation with 1 mL of A reagent for 10 min on ice. Next, cells were homogenized on ice using a 7 mL Dounce tissue grinder (40 strokes). The homogenates were centrifuged at 1,000 × *g* and 4,000 *× g* for 10 min at 4°C. Thereafter, supernatants were centrifuged at 17,000 × *g* for 30 min at 4°C. The pellet was resuspended in B reagent (500 µL) and centrifuged again at 17,000 × *g* for 30 min. The pellet was suspended in C reagent, followed by incubation for 30 min and centrifugation at 12,000 × *g* for 15 min. Lysosomal protein-containing supernatants were used for Western blotting.

### Statistical analysis

Data processing and statistical analysis were performed using Fiji and GraphPad Prism software. Results from triplicate experiments are presented as mean ± SD, with statistical significance assessed using an unpaired *t*-test. Significance is indicated as follows: ns, not significant, *P < 0.05, **P < 0.01, ***P < 0.001, and ****P < 0.0001.

## Data Availability

Underlying data and the accession numbers are available in the main text. All other raw data will be shared upon reasonable request.

## SUPPLEMENTAL MATERIAL

Supplemental figures mbio.02903-25-s0001.docx Fig. S1 to S15.

ASM does not own the copyrights to Supplemental Material that may be linked to, or accessed through, an article. The authors have granted ASM a non-exclusive, world-wide license to publish the Supplemental Material files. Please contact the corresponding author directly for reuse.
