# Supplementary material for: Progranulin-driven lysosomal acidification facilitates exocytosis of PHEV-hijacked lysosomes for viral release
Source: mBio. 2025 Nov 25;17(1):e02903-25. doi: 10.1128/mbio.02903-25 (PMC12802303; doi:10.1128/mbio.02903-25)
Supplement: Supplemental figures — Fig. S1 to S15. [file mbio.02903-25-s0001.docx]

**Supporting information**

**Progranulin-driven lysosomal acidification facilitates exocytosis of PHEV-hijacked lysosomes for viral release**

Zhenzhen Wang, Yuzhu Chen, Wenqi He, Yuzhu Liu, Yubo Jiao, Gaili Wang, Jiyu Guan, Kui Zhao, Qiaoling Zhang, Feng Gao, Zi Li, Yungang Lan

**
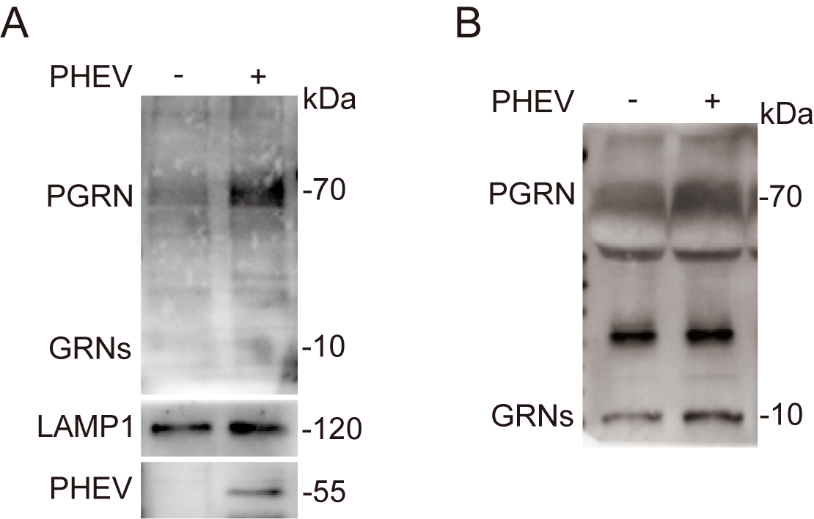
**

**FIG S1** (A) The protein levels of PGRN, GRNs, LAMP1 and PHEV in the isolated lysosomes from mock or PHEV-infected cells at 48 hpi, respectively. (B) The protein levels of PGRN and GRNs in the extracellular medium from mock or PHEV-infected cells at 48 hpi, respectively.


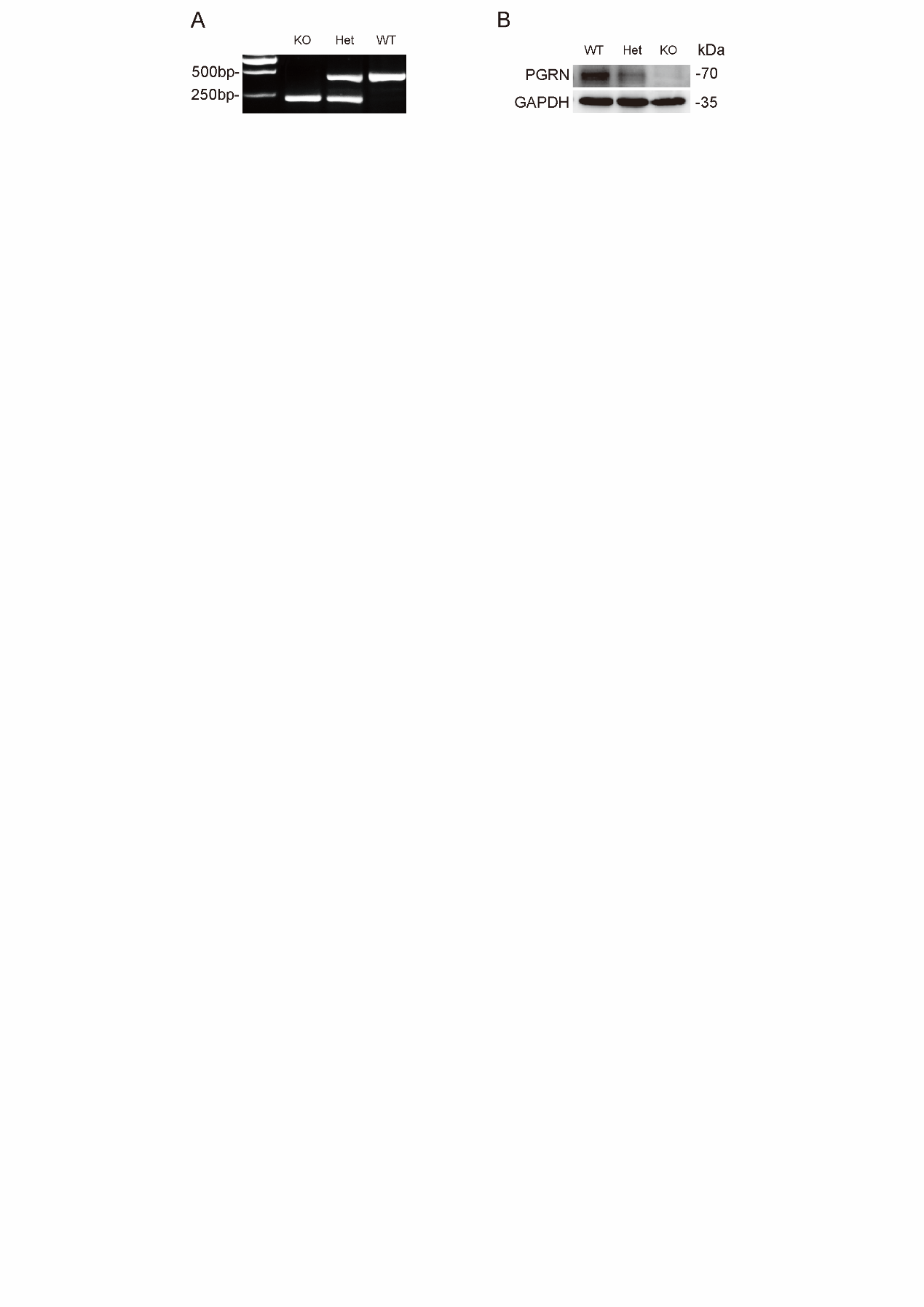
**FIG S2** Ablation of PGRN gene product was confirmed by PCR (A) and western blot analysis using antibodies specific to PGRN (B).


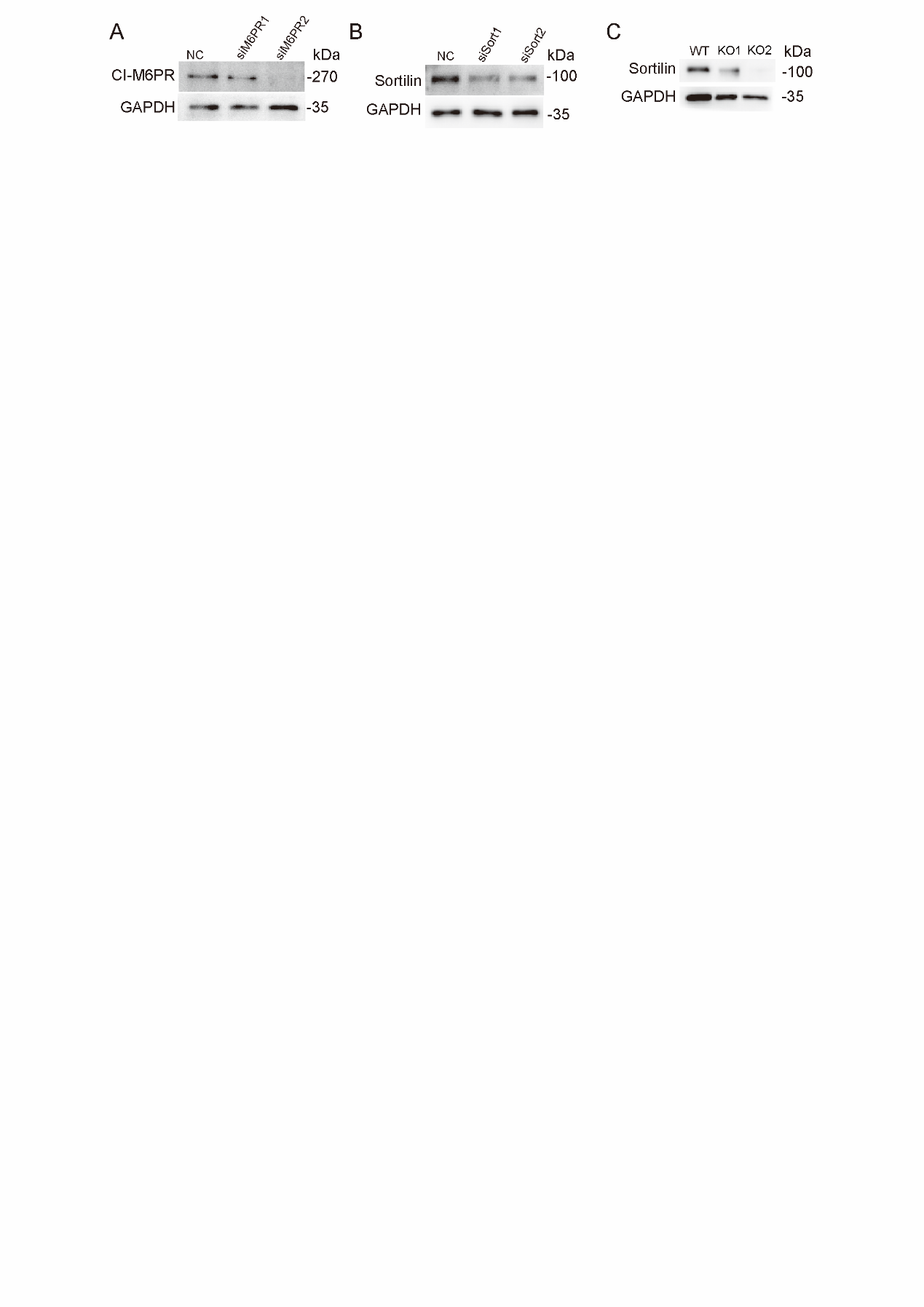


**FIG S3** **Effects of different proteins knockout or knockdown.**

(A) The CI-M6PR protein expression in scrambled siRNA (NC)- or siM6PR-treated N2a cells. (B) The sortilin protein expression in scrambled siRNA (NC)- or siSort-treated N2a cell. (C) The sortilin protein expression in WT or sortilin KO cells. Experiments were repeated three times.

**
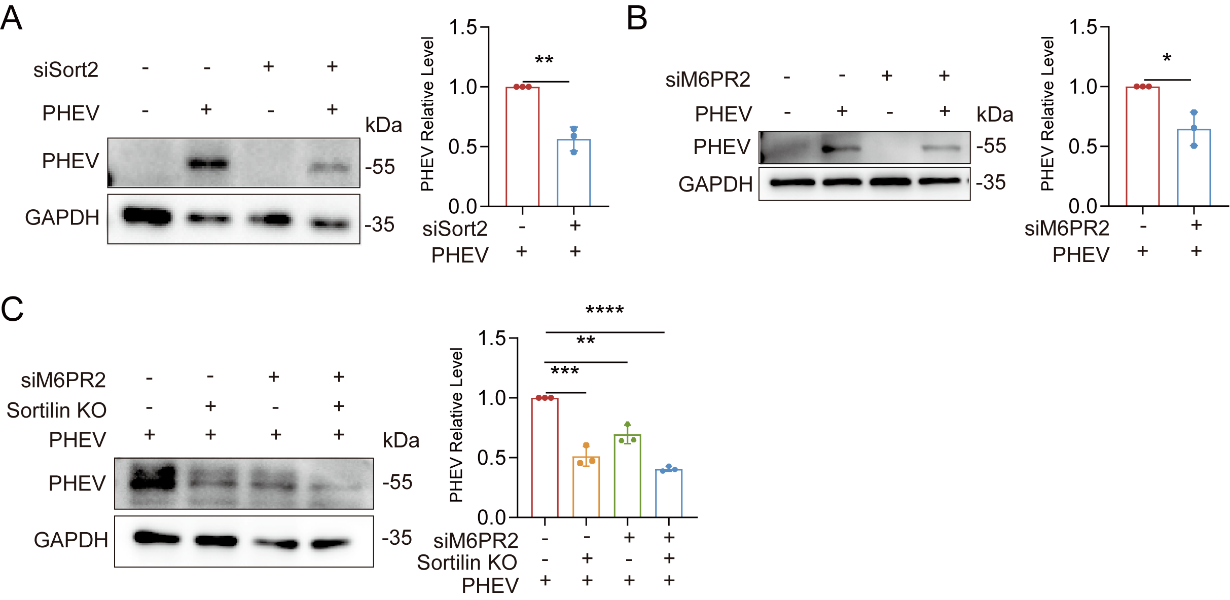
**

**FIG S4** **The impact of Sortilin and C1-M6PR on PHEV replication.**

(A) The protein levels of PHEV N and GAPDH were analyzed by western blotting in mock or PHEV-infected WT or siSort2-treated cells. (B) The protein levels of PHEV N and GAPDH were analyzed by western blotting in mock or PHEV-infected WT or siM6PR2-treated cells. (C) The protein levels of PHEV N and GAPDH were analyzed by western blotting in PHEV-infected WT cells, PHEV-infected sortilin KO cells, PHEV-infected siM6PR2-treated cells, and PHEV-infected siM6PR2-treated sortilin KO cells. Experiments were repeated three times. Representative blots are shown. Data are shown as mean ± SD. P values were considered significant when p < 0.05 and denoted as, *, p < 0.05, **, p < 0.01, ***, p < 0.001, ****, p < 0.0001.


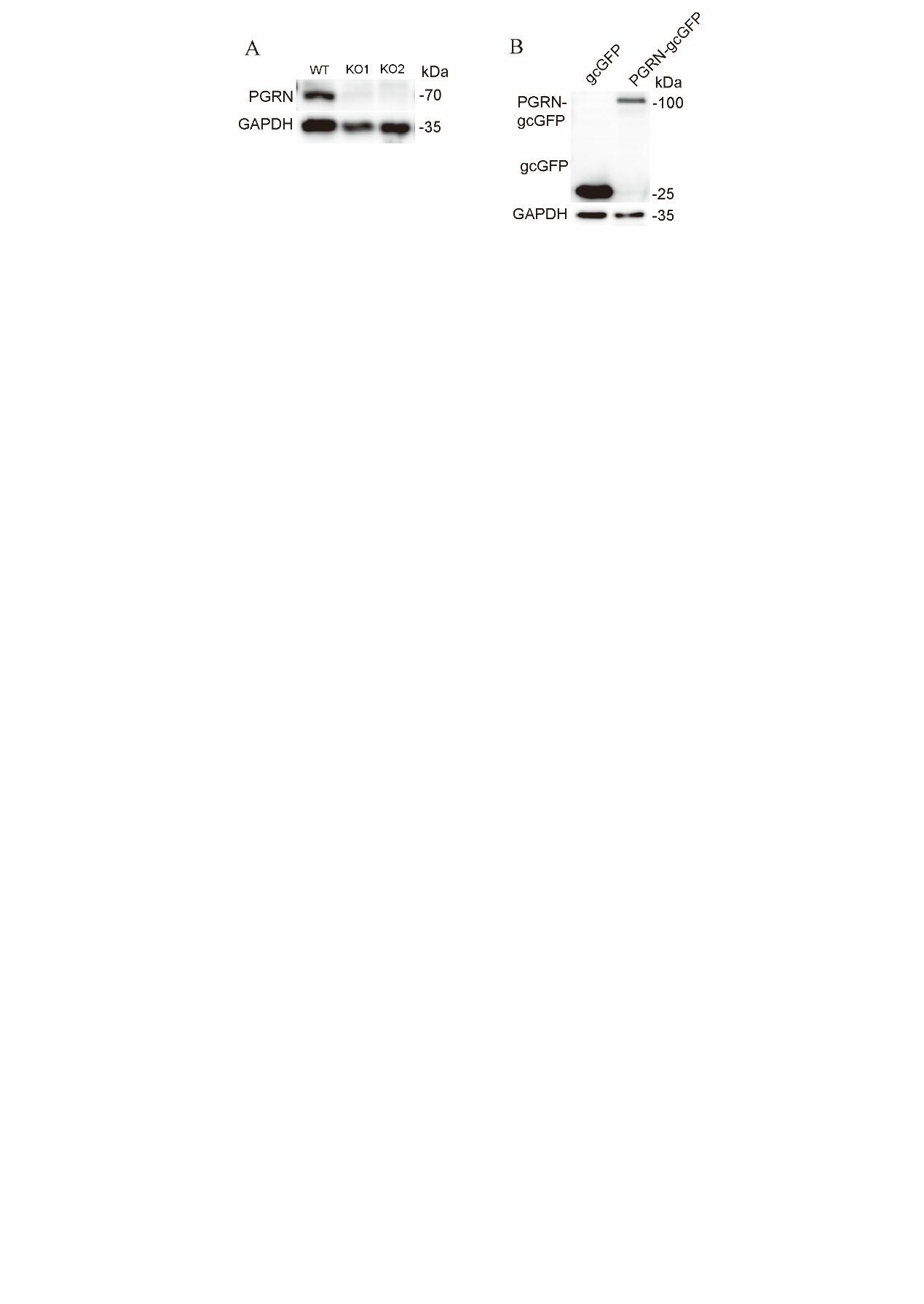
**FIG S5** **Validation of PGRN knockout and overexpression cell lines.**

(A) PGRN protein expression in WT or PGRN KO N2a cells. (B) The PGRN-gcGFP protein expression in PGRN-gcGFP- or gcGFP-overexpressing cells.


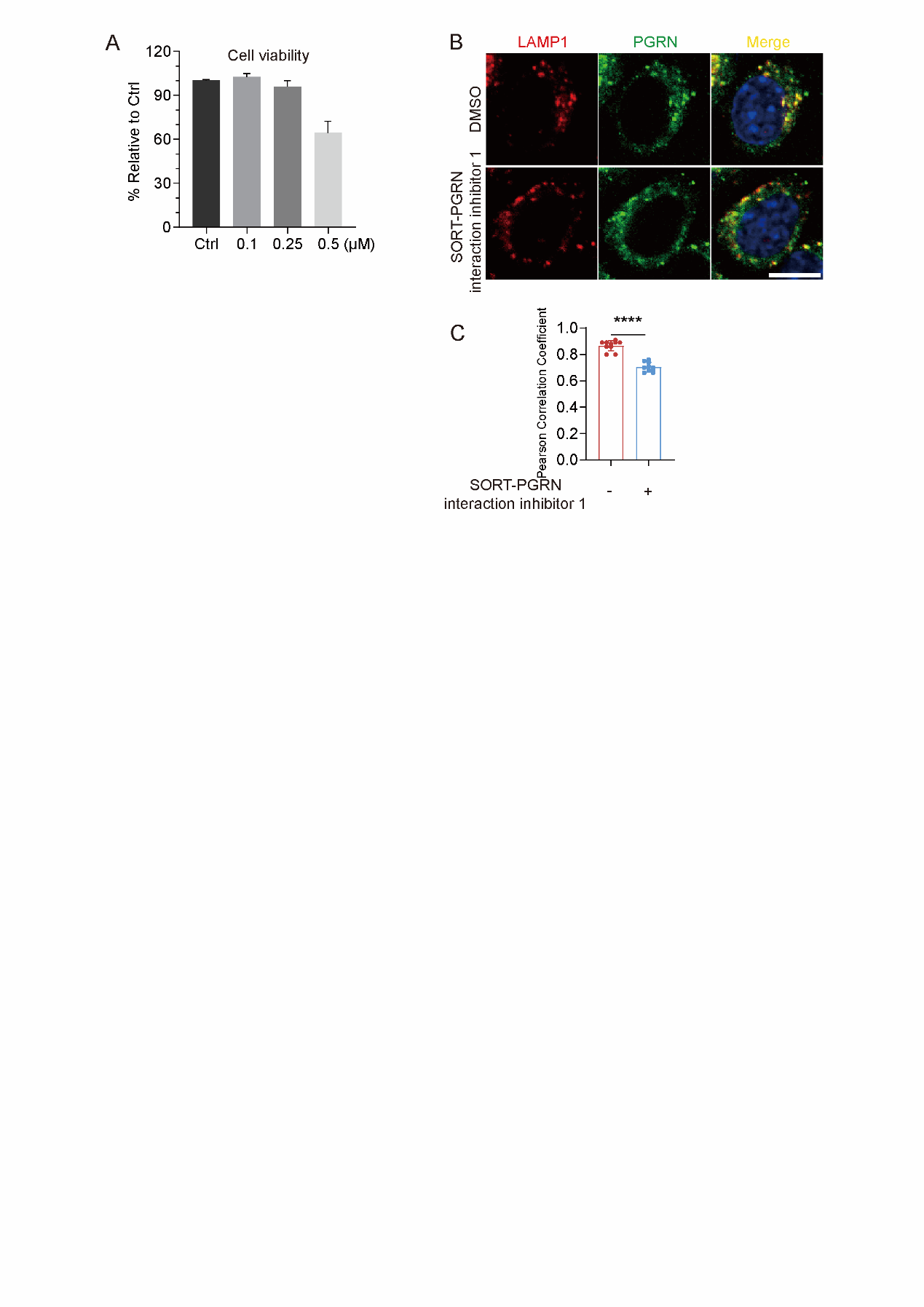


**FIG S6** **Effect of SORT-PGRN interaction inhibitor 1 on PGRN lysosomal trafficking.**

(A) CCK-8 assays were performed on N2a cells treated different concentrations of SORT-PGRN interaction inhibitor 1 for 24 h to assess the cytotoxicity of SORT-PGRN interaction inhibitor 1. (B) Colocalization of LAMP1 (red) with PGRN (green) in N2a cells with DMSO or 250 nM SORT-PGRN interaction inhibitor 1 treatment. Scale bar: 10 μm. (C) The Pearson’s correlation coefficients of the images between LAMP1 and PGRN were analyzed by using Image J (n=10 cells). Experiments were repeated three times. Representative images are shown. Data are shown as mean ± SD. P values were considered significant when p < 0.05 and denoted as, ****, p < 0.0001.


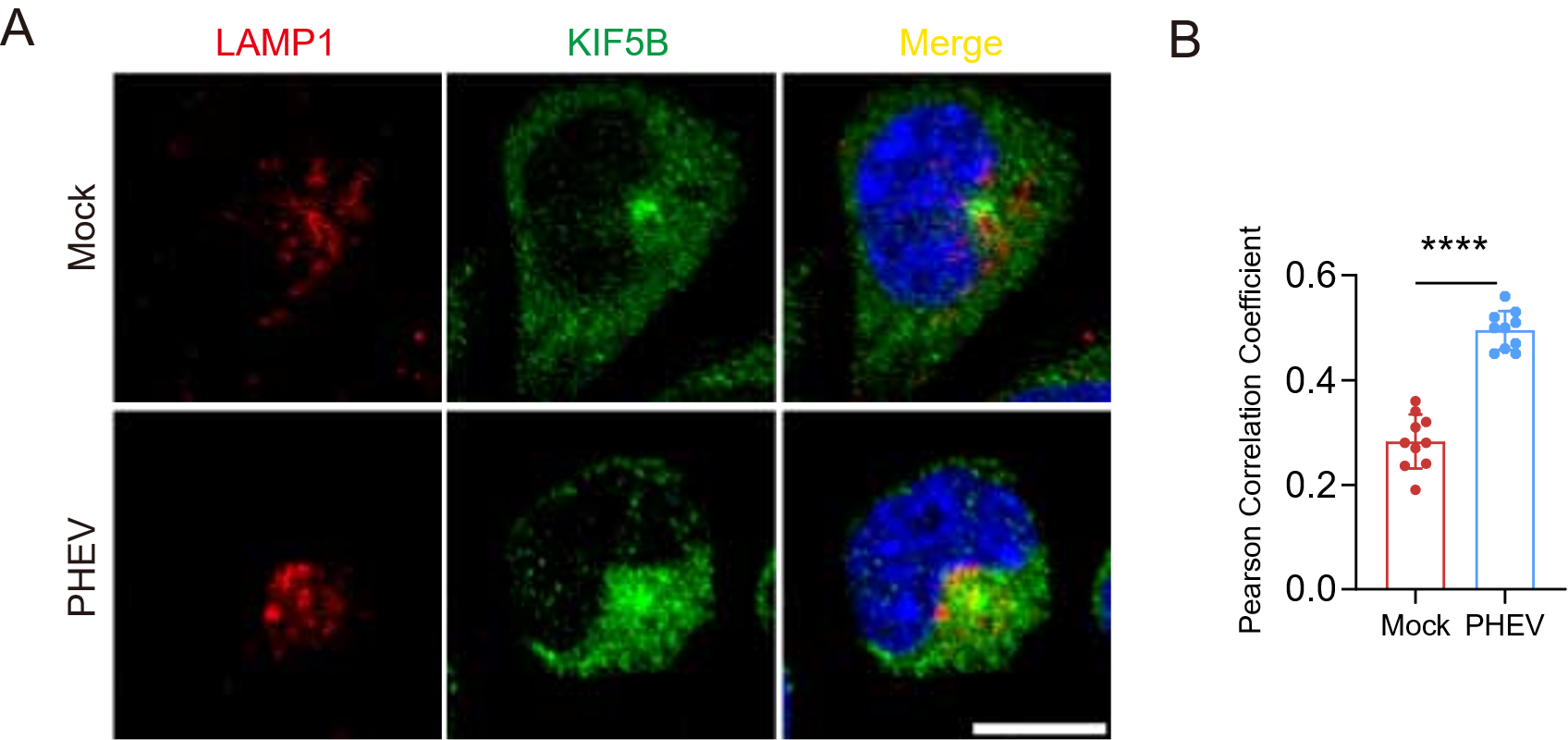


**FIG S7** **The effect of PHEV infection on KIF5B recruitment to lysosomes.**

(A) Colocalization of LAMP1 (red) with KIF5B (green) in mock- or PHEV-infected N2a cells. Scale bar: 10 μm. (B) The Pearson’s correlation coefficients of the images between LAMP1 and KIF5B were analyzed by using Image J (n=10 cells). Experiments were repeated three times. Representative images are shown. Data are shown as mean ± SD. P values were considered significant when p < 0.05 and denoted as, ****, p < 0.0001.


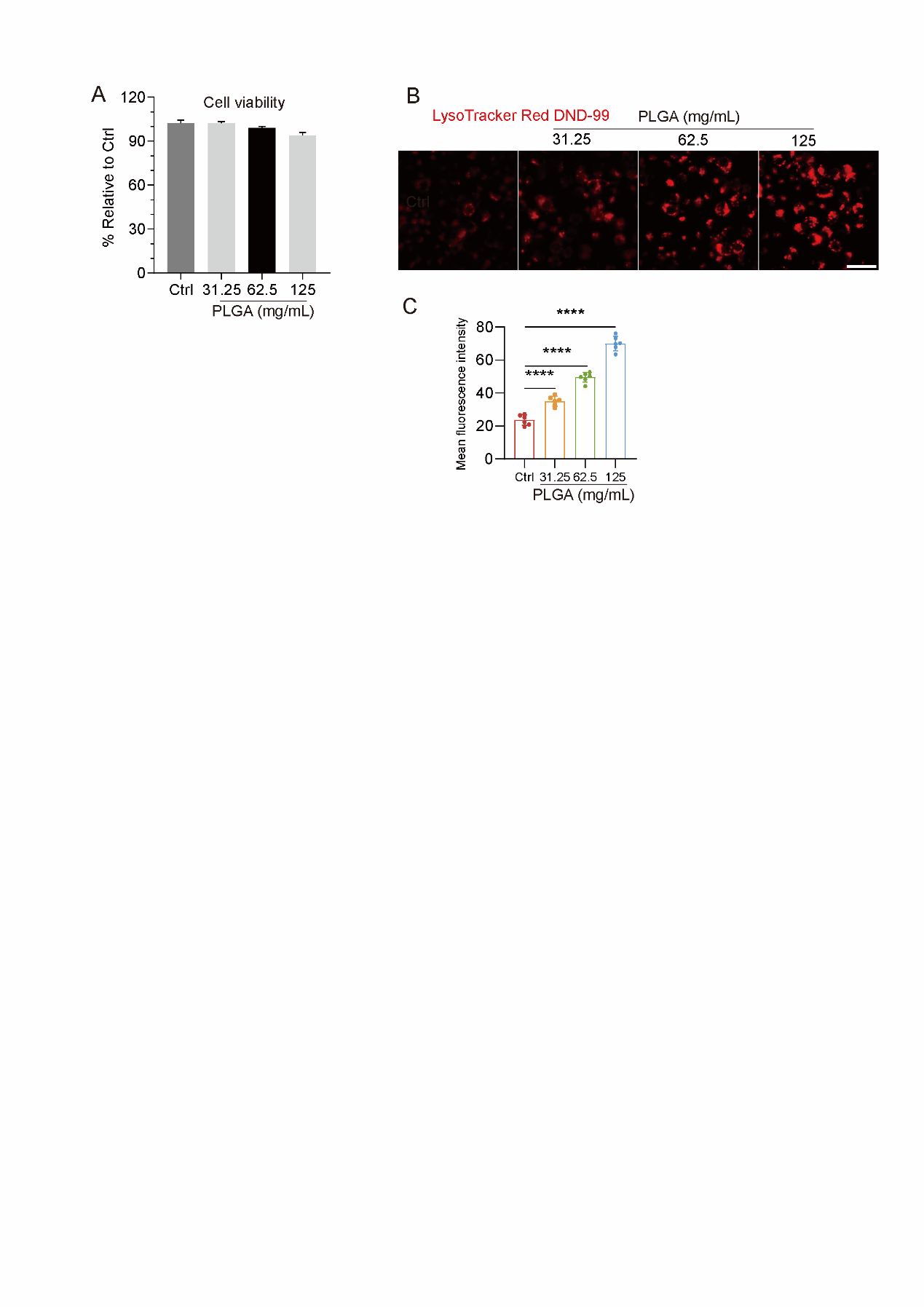


**FIG S8 Cell viability and lysosomal acidification of N2a cells with various PLGA concentrations (31.25, 62.5, 125 mg/mL) for 24 h.**

(A) CCK-8 assays were performed on N2a cells treated with different concentrations of PLGA for 24 h to assess the cytotoxicity of PLGA. (B) The different concentrations of PLGA-treated N2a cells were stained by LysoTracker Red DND-99. Scale bar, 40 μm. (C) Quantification of LysoTracker Red DND-99 fluorescence intensity. Experiments were repeated three times. Data are shown as mean ± SD. P values were considered significant when p < 0.05 and denoted as, ****, p < 0.0001.


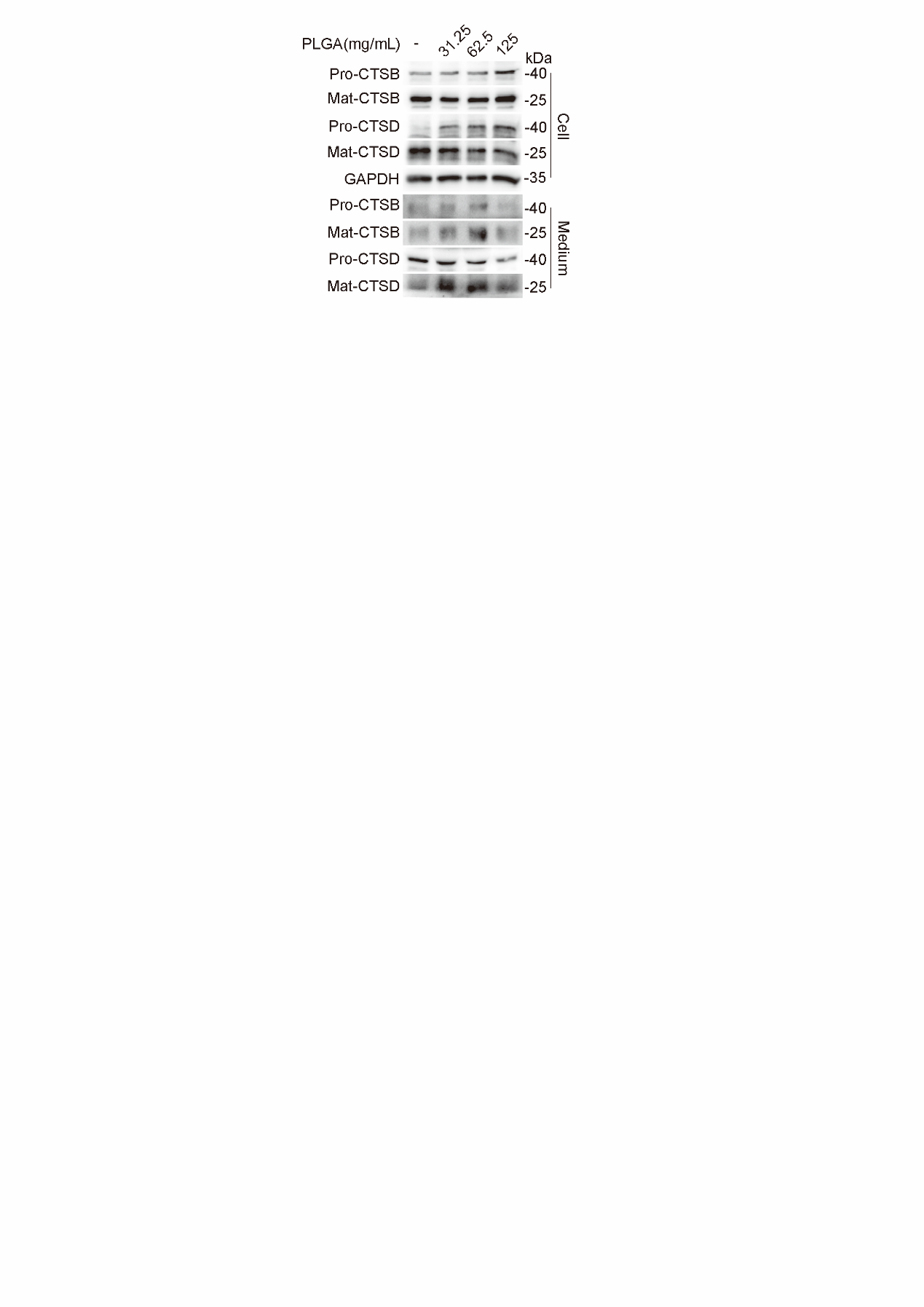


**FIG S9 Effects of PLGA on the expression and secretion of lysosomal hydrolase.**

The protein levels of Pro-CTSD, Pro-CTSB, Mat-CTSD, Mat-CTSB, and GAPDH in different concentrations of PLGA-treated N2a cells were analyzed by western blotting, respectively. Experiments were repeated three times.


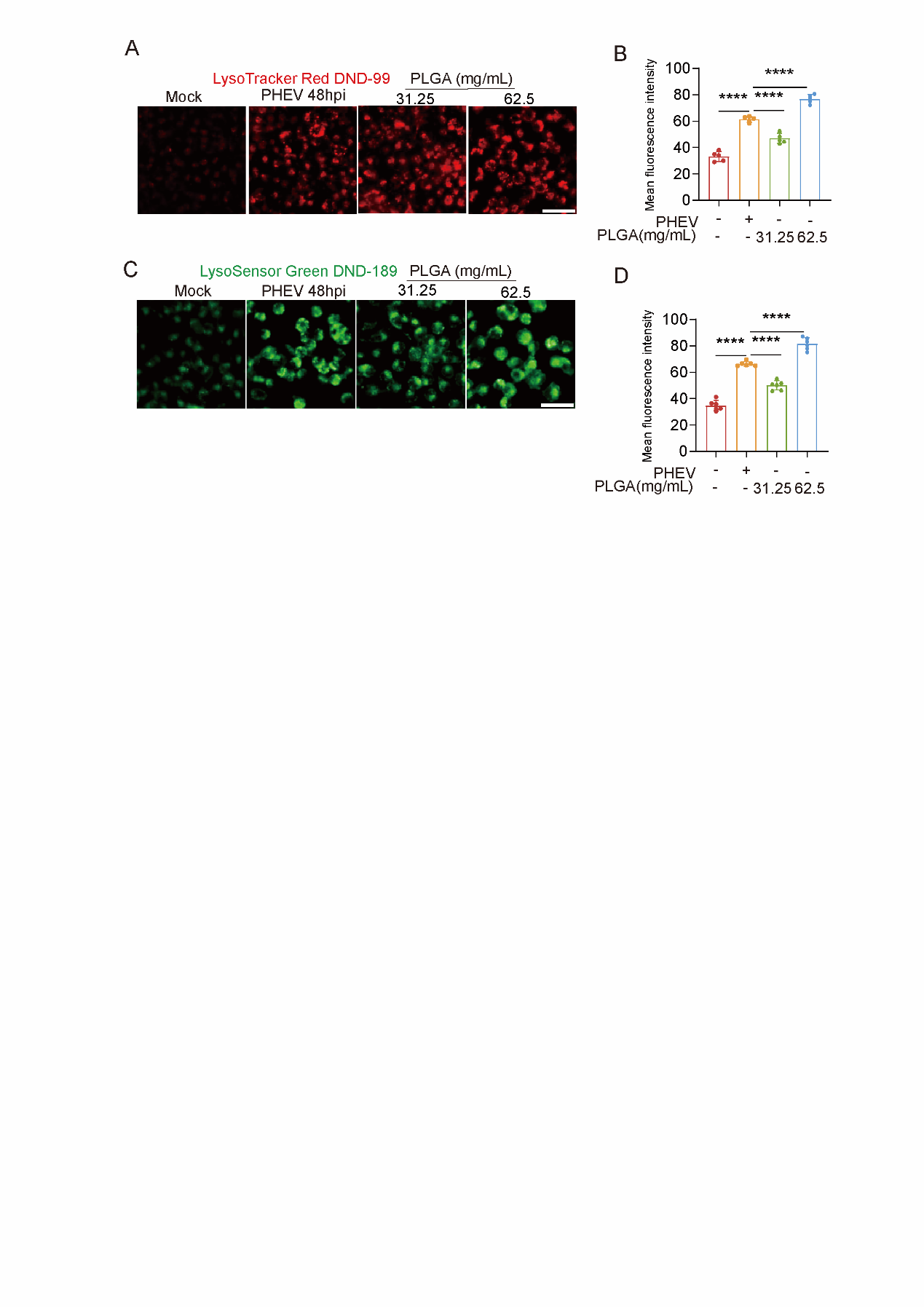


**FIG S10** **The lysosomal acidity observed in PHEV-infected N2a cells at 48 hpi fell within the range of lysosomal acidity induced by 31.25-62.5 mg/mL PLGA treatment of N2a cells.**

(A) PHEV-infected N2a cells at 48 hpi or the different concentrations of PLGA-treated N2a cells for 24 h were stained by LysoTracker Red DND-99. Scale bar, 40 μm. (B) Quantification of LysoTracker Red DND-99 fluorescence intensity. (C) PHEV-infected N2a cells at 48 hpi or the different concentrations of PLGA-treated N2a cells for 24 h were stained by LysoSensor Green DND-189. Scale bar, 40 μm. (D) Quantification of LysoSensor Green DND-189 fluorescence intensity. The above experiments were repeated three times. Representative blots and images are shown. Data are shown as mean ± SD. P values were considered significant when p < 0.05 and denoted as, ****, p < 0.0001.


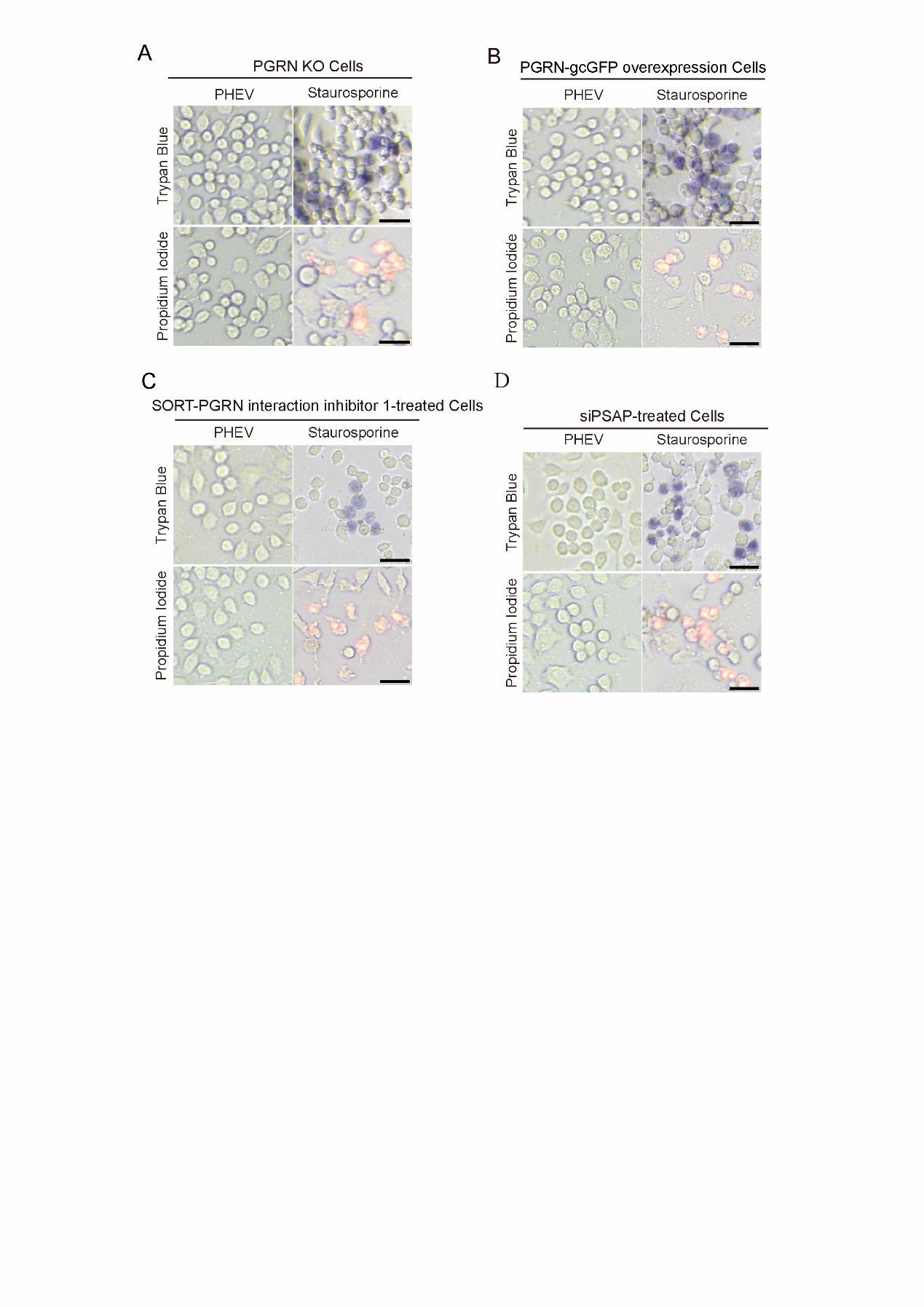


**FIG S11** **The changes in plasma membrane permeability under our experimental conditions.**

(A-D) Trypan blue and propidium iodine exclusion were used to detect changes in plasma membrane permeability in PHEV-infected PGRN KO, PGRN-overexpressing cells, SORT-PGRN interaction inhibitor 1 and siPSAP-treated cells at 48 hpi, respectively. Staurosporine-treated cells were seen as a positive control of cell membrane rupture. Scale bar, 40 μm. Experiments were repeated three times.


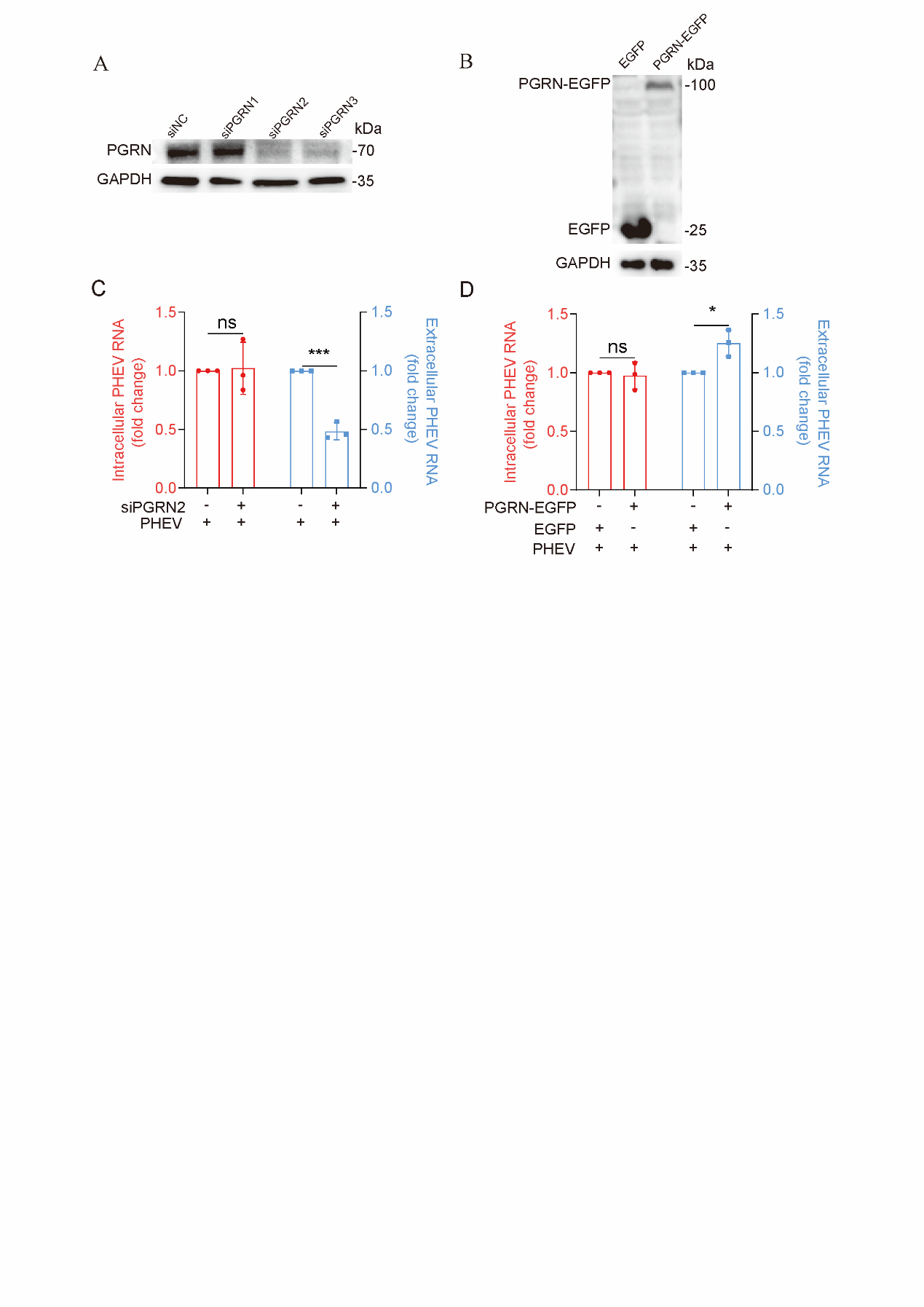


**FIG S12** **The effect of PGRN on PHEV release under conditions of PGRN knockdown and transient PGRN-EGFP overexpression.**

(A) PGRN protein level in N2a cells with NC or siPGRN treatment. (B) PGRN-EGFP protein level in N2a cells with PGRN-EGFP transfection. (C and D) The PHEV N genomic RNA was determined using qPCR in PHEV-infected PGRN knockdown or transient PGRN-EGFP overexpression cells at 48 hpi, respectively. Experiments were repeated three times. Representative blots and images are shown. Data are shown as mean ± SD. P values were considered significant when p < 0.05 and denoted as, *, p < 0.05, ***, p < 0.001, ns, not significant.


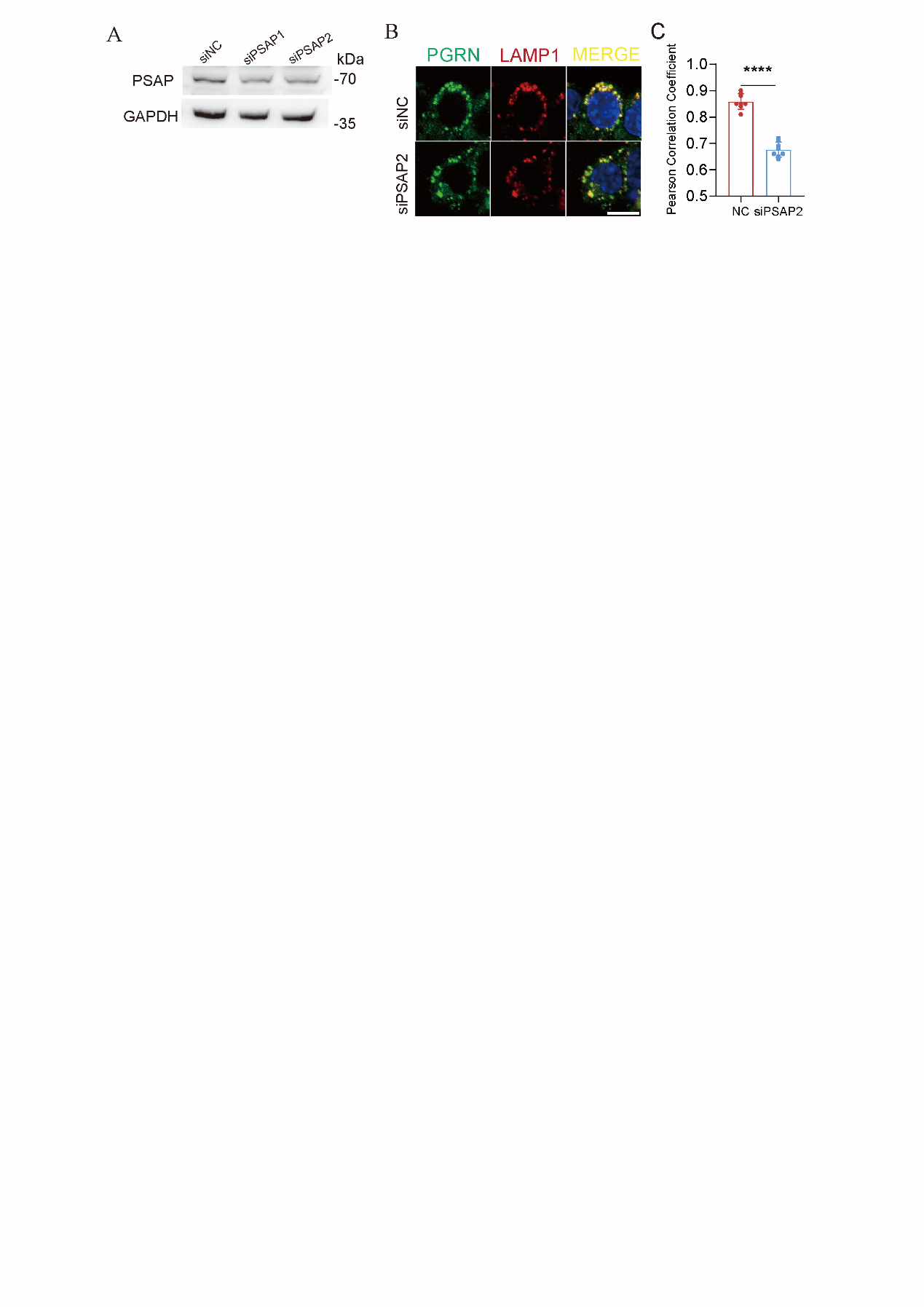


**FIG S13** **Effect of siPSAP on PGRN lysosomal trafficking.**

(A) PSAP protein level in N2a cells with NC- or siPSAP treatment. (B) Colocalization of LAMP1 (red) with PGRN (green) in NC- or siPSAP-treated N2a cells. Scale bar: 10 μm. (C) The Pearson’s correlation coefficients of the images between LAMP1 and PGRN were analyzed by using Image J (n=8 cells). Experiments were repeated three times. Representative blots and images are shown. Data are shown as mean ± SD. P values were considered significant when p < 0.05 and denoted as, ****, p < 0.0001.


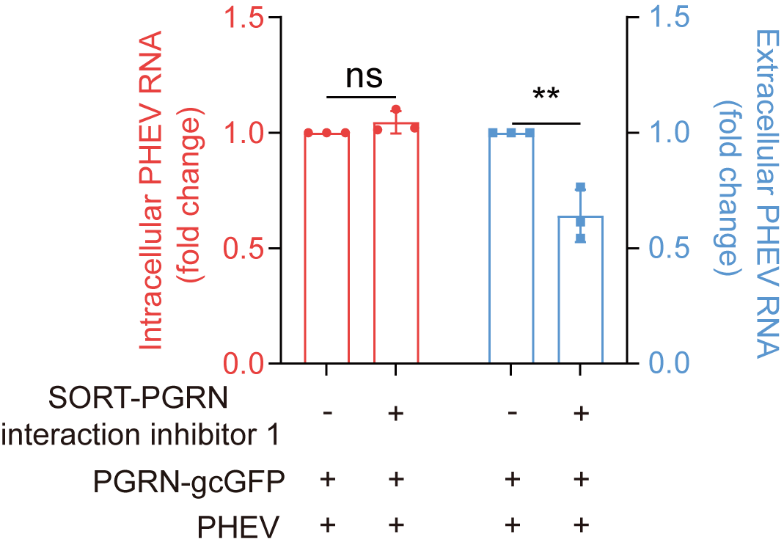


**FIG S14** The PHEV N genomic RNA was determined using qPCR in PHEV-infected SORT-PGRN interaction inhibitor 1-treated PGRN-gcGFP-overexpressing cells at 48 hpi.


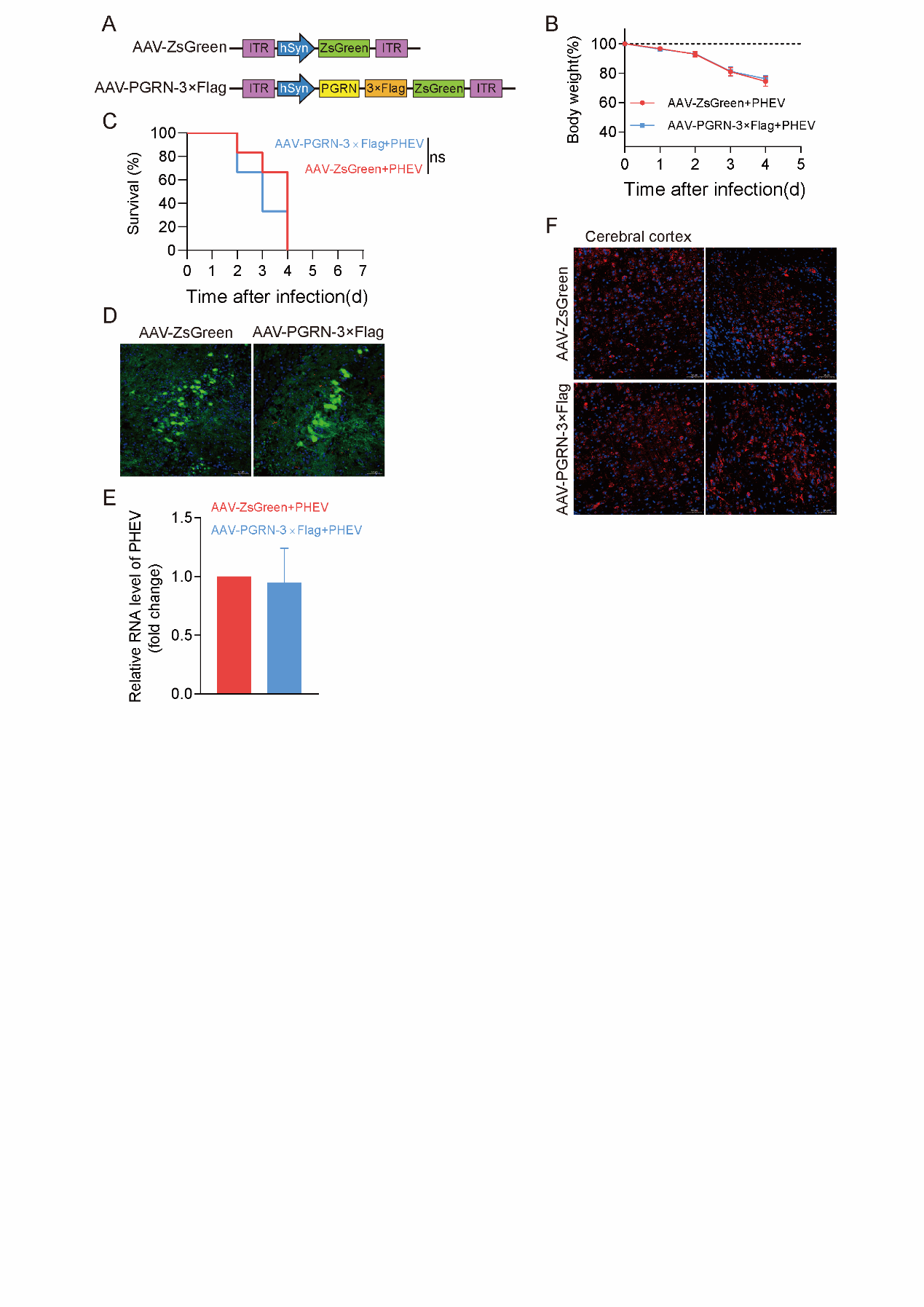


**FIG S15 PGRN overexpression has no effect on the PHEV neural transmission in the central nervous system.**

(A) AAV-ZsGreen or AAV-PGRN-3xFlag construct. (B) Changes in body weights of mice in each treatment group were monitored daily. (C) Kaplan-Meier survival curve showing survival time of PHEV-infected mice pretreated AAV-ZsGreen or AAV-PGRN-3xFlag (n=6). (D) High magnification immunostaining for ZsGreen in CA2 hippocampal tissue derived from mice treated with AAV-ZsGreen or AAV-PGRN-3xFlag. (E) PHEV N genomic RNA levels in PHEV-infected AAV-ZsGreen- or AAV-PGRN-3xFlag-pretreated mice brain. (F) PHEV-infected AAV-ZsGreen- or AAV-PGRN-3xFlag-pretreated mice brain sections were immunostained with anti-PHEV (red) antibodies. The above experiments were repeated three times. Representative blots and images are shown. Data are shown as mean ± SD. P values were considered significant when p < 0.05 and denoted as, ns, not significant.
